# Genome-wide identification of the *SnRK2* gene family and its response to drought stress in *Bombax ceiba*

**DOI:** 10.3389/fpls.2026.1873223

**Published:** 2026-07-07

**Authors:** Yumei Shi, Zhifang Zhang, Ruoxin He, Liangju He, Yongxiu Wang, Wenjie Ma, Zhenghao Yan, Youqiang Tao, Zhi Yang, Changxin Luo

**Affiliations:** College of Biology and Food Engineering, Qujing Normal University, Qujing, Yunnan, China

**Keywords:** ABA signaling, *Bombax ceiba*, drought stress, genome-wide identification, *SnRK2* gene family, tissue-specific expression

## Abstract

**Introduction:**

Drought is a major abiotic stress limiting the growth and ecological adaptation of tropical and subtropical trees. The *SnRK2* gene family is a core regulator in ABA signaling and drought response pathways. However, genome-wide identification and functional characterization of the SnRK2 family remain unclear in *Bombax ceiba*, a typical drought-tolerant tropical pioneer tree species with important ecological and economic value.

**Methods:**

We performed genome-wide identification of the *BcSnRK2* gene family using bioinformatics approaches. Phylogenetic relationships, gene structures, conserved motifs, cis-acting elements, chromosomal localization, and protein structures were systematically analyzed. Subcellular localization was verified by transient expression in *Nicotiana benthamiana*. Tissue-specific expression and drought-responsive patterns were detected by qRT-PCR under 10% PEG6000 treatment. Protein–protein interaction networks were predicted using the STRING database.

**Results:**

A total of nine *BcSnRK2* genes were identified and unevenly distributed across eight chromosomes. All BcSnRK2 proteins contained conserved kinase domains and shared a highly conserved exon–intron structure. Promoter regions harbored abundant ABA-responsive and stress-related cis-elements. *BcSnRK2* genes exhibited distinct tissue-specific expression profiles. All genes were significantly induced by drought stress in a tissue- and time-dependent manner, with *BcSnRK2.9* and *BcSnRK2.7* showing strong and sustained activation in shoots and *BcSnRK2.7* and *BcSnRK2.3* responding prominently in roots. BcSnRK2 proteins were localized in the cytoplasm, plasma membrane, and nucleus, and were predicted to interact with core components of the ABA signaling pathway.

**Discussion:**

The BcSnRK2 family exhibits evolutionary conservation and functional divergence in *Bombax ceiba*. The compact size of the SnRK2 family, conserved structural features, and distinct tissue-specific drought response patterns are consistent with a streamlined stress signaling system that may contribute to the ecological adaptation of *Bombax ceiba* in seasonally dry tropical environments, although formal evolutionary analyses are required to establish adaptive significance. This study provides valuable gene resources for drought resistance breeding of woody plants and advances the understanding of stress signaling mechanisms in tropical trees.

## 1 Introduction

Drought stress is one of the most widespread and destructive abiotic stresses limiting global agricultural production, forest ecosystem stability, and plant geographical distribution. With the intensification of global climate change, the frequency, duration, and affected area of extreme drought events have increased significantly, leading to serious losses in crop yield, forest degradation, and ecological environment deterioration (Rennenberg et al., 2006; Fahad et al., 2017; Xiao et al., 2023; Nie et al., 2023). Unlike animals, plants are sessile organisms that cannot escape adverse environments; instead, they have evolved a series of sophisticated and efficient molecular, cellular, and physiological mechanisms to perceive, transduce, and respond to drought stress signals (Zhu, 2016; Chen et al., 2026). These mechanisms mainly include signal perception and transduction, transcriptional regulation, stress-responsive gene expression, osmotic adjustment, reactive oxygen species scavenging, and stomatal movement regulation (Shinozaki and Yamaguchi-Shinozaki, 2007). At the molecular level, protein phosphorylation and dephosphorylation mediated by protein kinases and phosphatases constitute one of the core rapid response systems for plants to cope with drought stress. Among them, the sucrose non-fermenting-1-related protein kinase 2 (SnRK2) family is considered as a central hub connecting abscisic acid (ABA) signal pathway and drought stress response, which plays an irreplaceable role in plant adaptation to water deficit environment (Lin et al., 2021; Horvat et al., 2024; Michalak et al., 2026).

SnRK2 kinases constitute a plant-specific family of serine/threonine (Ser/Thr) protein kinases. Structurally, each SnRK2 protein comprises three functional domains: an N-terminal adenosine triphosphate (ATP)-binding domain, a central conserved Ser/Thr kinase domain, and a variable C-terminal regulatory domain (Saha et al., 2014; Holappa et al., 2017; Shinozawa et al., 2019). The N-terminal kinase domain is responsible for ATP binding and substrate phosphorylation, while the C-terminal domain is mainly involved in protein–protein interaction and response to ABA and osmotic stress signals (Kulik et al., 2011; Maszkowska et al., 2021). Based on amino acid sequence similarity, gene structure characteristics, and response patterns to ABA, SnRK2 family members can be divided into three distinct subgroups: Group I, Group II, and Group III (Kulik et al., 2011). A large number of studies have confirmed that Group III members are strongly activated by ABA and are the core kinases in ABA-dependent drought stress signaling pathway; Group I and Group II members are not sensitive or weakly sensitive to ABA, but can be rapidly induced and activated by osmotic stress such as drought, high salt, and low temperature, participating in ABA-independent stress response pathways (Yoshida et al., 2002; Krzywińska et al., 2016; Soma et al., 2020; Jin et al., 2025). Through phosphorylating downstream transcription factors (such as ABF/AREB, DREB, NAC) and functional proteins (such as ion channel proteins, aquaporins, antioxidant enzymes), SnRK2 kinases can regulate the expression of a large number of stress-responsive genes, promote stomatal closure, reduce water loss, enhance osmotic protection ability, and ultimately improve plant drought tolerance (Kobayashi et al., 2004; Fujii et al., 2007; Acharya et al., 2013; Mao et al., 2020).

In recent years, with the rapid development of high-throughput sequencing technology and bioinformatics analysis methods, genome-wide identification and functional analysis of *SnRK2* gene family have been successively completed in a variety of model plants, crops, and horticultural plants (Wang et al., 2015; Yoo et al., 2016; Hussain et al., 2022; Mathura et al., 2023; Xu and Wang, 2025; Tang et al., 2025). In *Arabidopsis thaliana*, 10 SnRK2 family members (AtSnRK2.1–AtSnRK2.10) have been identified, among which AtSnRK2.2, AtSnRK2.3, and AtSnRK2.6 (OST1) are the key kinases in ABA signal transduction, regulating stomatal closure and seed germination (Lin et al., 2021). In rice (Oryza sativa), 10 *OsSnRK2* genes have been found, and some of them have been proven to be involved in drought, salt, and cold stress responses (Zhong et al., 2020; Yu et al., 2022). In addition, genome-wide identification of SnRK2 family has also been reported in maize (Huai et al., 2008), wheat (Jiang et al., 2022), cotton (Liu et al., 2017), soybean (Zhao et al., 2017), euphratica (Jin et al., 2025), sugarcane (Li et al., 2017), and other species. These studies have shown that the number, structure, and function of SnRK2 family members are relatively conserved in different plants, but there are also obvious species-specific differences in gene expansion, expression patterns, and stress response modes, which are closely related to plant genome size, evolutionary history, and environmental adaptability. However, up to now, there is still a lack of systematic and in-depth genome-wide analysis of *SnRK2* gene family in woody plants with strong drought resistance, especially in tropical and subtropical representative tree species, which limits our understanding of the molecular mechanism of drought resistance in woody plants.

*Bombax ceiba* Linn. commonly known as kapok or red cotton tree, is a large deciduous arbor belonging to the Malvaceae family, which is widely distributed in tropical and subtropical regions of Asia (Gao et al., 2018; Amat-Ur-Rasool et al., 2020). As a typical pioneer tree species in arid and semi-arid habitats, *Bombax ceiba* has strong adaptability to drought, barren, high temperature, and other adverse environments, and plays an important role in windbreak and sand fixation, water and soil conservation, ecological restoration, and landscape construction (Luo et al., 2021, 2024; Yasin et al., 2026). In addition, *Bombax ceiba* has high economic value, its fiber can be used as filling material, its flower can be used as medicine and food, its wood can be used for papermaking and furniture manufacturing, and it also has important ornamental and cultural value (Jain and Verma, 2012; Komati et al., 2022; Kumari et al., 2025). At present, the research on *Bombax ceiba* mainly focuses on morphological characteristics, physiological and biochemical responses, cultivation techniques, and resource investigation, while the research on molecular biology, especially the genome-wide identification and functional analysis of key gene families related to drought stress, is still in the initial stage. The completion of *Bombax ceiba* genome sequencing provides an important data foundation for us to carry out genome-wide analysis of functional gene families and explore the molecular mechanism of drought resistance (Zhou et al., 2015; Gao et al., 2018; Shao et al., 2024; Yuan et al., 2025).

The SnRK2 gene family functions as a central regulatory hub in ABA signaling and plant drought stress responses. Although genome-wide characterization of SnRK2 families has been extensively reported in model herbaceous species and major crops, systematic identification and functional analysis of *SnRK2* genes in drought-adapted tropical woody plants remain limited. *Bombax ceiba*, a highly drought-tolerant pioneer tree species widely distributed in tropical and subtropical dry valleys, possesses unique ecological adaptability to seasonally arid environments, yet its *SnRK2* gene family has not been systematically investigated. In this study, we performed a comprehensive genome-wide identification of the *BcSnRK2* gene family, followed by systematic analyses of phylogenetic relationships, gene structure, conserved motifs, promoter cis-acting elements, chromosomal distribution, and protein architecture. We further determined the tissue-specific expression patterns of all *BcSnRK2* members and their dynamic transcriptional responses to PEG-simulated drought stress, verified the subcellular localization of BcSnRK2 proteins, and predicted their protein interaction networks associated with ABA signaling. Our results reveal a compact SnRK2 family comprising nine members in *Bombax ceiba*, with pronounced structural conservation and clear tissue-specific functional differentiation among paralogs. Notably, we identified distinct temporal and tissue-specific drought response patterns, with typical ABA-insensitive clade members exhibiting significant drought inducibility. These findings provide fundamental genetic resources and reference data for exploring drought adaptation mechanisms and genetic improvement of stress resistance in woody plants.

## 2 Materials and methods

### 2.1 Plant materials and drought stress treatment

Seeds of *Bombax ceiba* were collected from a hot and dry valley (latitude 25°40′50.06″ N, longitude 101°53′27.76″ E). The seeds were surface-sterilized by immersion in 30% hydrogen peroxide solution for 30 minutes, followed by five washes with sterile water. To initiate germination, the disinfected seeds were placed on sterile gauze in 15-cm Petri dishes and cultivated at room temperature. The seeds were rinsed twice daily with sterile water. After germination, the seedlings were transferred to cultivation pots filled with sterilized perlite and grown at 25 °C under a 16-hour light/8-hour dark photoperiod for three weeks (Luo et al., 2024).

For drought stress treatment, seedlings were transferred to a 10% (w/v) polyethylene glycol 6000 (PEG6000, Sangon Biotech, Shanghai, China) solution to simulate osmotic/drought stress. This concentration was chosen based on preliminary experiments showing that 10% PEG6000 induced visible leaf wilting after 24 h, allowing us to capture early transcriptional responses of *BcSnRK2* genes to drought signals rather than severe stress responses. Upper tissues (including leaves and young stems) and lower tissues (including main roots and basal stems) were harvested separately at 0, 1, 3, 6, 12, and 24 hours after treatment. For each time point and tissue type, three independent biological replicates were collected, each consisting of pooled tissues from three randomly selected seedlings to minimize the influence of inter-individual genetic variation. All samples were immediately frozen in liquid nitrogen and stored at −80 °C until RNA extraction. The 0-hour samples (untreated) served as the control.

### 2.2 RNA extraction and quantitative real-time PCR

Total RNA was extracted from frozen tissue samples using the Huayueyang RNA extraction kit (Huayueyang, China) according to the manufacturer’s instructions. RNA integrity was assessed by 1% (w/v) agarose gel electrophoresis, and RNA concentration and purity were measured with an Eppendorf BioPhotometer^®^ D30 (Eppendorf, Germany). Only RNA samples with A260/A280 ratios between 1.9 and 2.1 and A260/A230 ratios >2.0 were used for downstream analysis. First-strand cDNA was synthesized from 1.5 μg of total RNA using the PrimeScript™ RT Reagent Kit with gDNA Eraser (Takara). The resulting cDNA was diluted 10-fold and stored at −20 °C.

qRT-PCR was performed using TB Green^®^ Premix Ex Taq™ II (Takara, Japan) on a LightCycler^®^ 96 (Roche). The reaction mixture (20 μL) contained 10 μL of TB Green Premix, 0.8 μL each of forward and reverse primers (10 μM) (**Supplementary Table 1**), 2 μL of diluted cDNA, and 6.4 μL of RNase-free water. The thermal cycling program was as follows: 95 °C for 30 s, followed by 40 cycles of 95 °C for 5 s and 60 °C for 30 s. Melting curve analysis was performed after the amplification cycles by gradually increasing the temperature from 60 °C to 95 °C (0.5 °C increment per 5 s) to confirm reaction specificity. Each reaction was performed in triplicate (technical replicates) for each of the three biological replicates. Relative expression levels were calculated using the 2^-^ΔΔCt method. The housekeeping gene *BcUBQ5* was used as the internal control (Li et al., 2022). A standard curve generated from a 10-fold dilution series of cDNA revealed an amplification efficiency of 94.69% for *BcUBQ5*, with an R² of 0.9998 (**Supplementary Figure 1**). The expression level in the control sample (0 h, lateral root for tissue-specific expression, or 0 h of each respective tissue for drought treatment) was set to 1.00.

### 2.3 Identification and physicochemical properties of BcSnRK2 family members

Genome and protein sequence data for *Bombax ceiba* were obtained from the National Center for Biotechnology Information (NCBI) database under BioProject accession number PRJNA429932 (Gao et al., 2018). The protein sequences of ten *Arabidopsis thalian*a SnRK2 members (AtSnRK2.1–AtSnRK2.10) were retrieved from The Arabidopsis Information Resource (TAIR, https://www.arabidopsis.org) and used as queries in BLASTP searches against the *Bombax ceiba* proteome (E-value < 1e^-5^). In parallel, for the preliminary identification of BcSnRK2 proteins, local BLASTP searches (E-value < 1e^-^²^0^) were performed using Hidden Markov Model (HMM) profiles of the SnRK2 conserved domains (Pfam: PF00069) obtained from the Pfam database (http://pfam.janelia.org/) (Jin et al., 2025). All candidate *SnRK2* gene sequences were subsequently verified using the SMART database (http://smart.embl-heidelberg.de/).

Physicochemical properties of the identified BcSnRK2 proteins, including amino acid length, molecular weight (MW), theoretical isoelectric point (pI), instability index, aliphatic index, and grand average of hydropathicity (GRAVY), were calculated using the ExPASy ProtParam tool (https://web.expasy.org/protparam/).

### 2.4 Phylogenetic analysis

Multiple sequence alignment of the full-length amino acid sequences of all BcSnRK2 and AtSnRK2 proteins was performed using MUSCLE implemented in MEGA 11 with default parameters. A maximum likelihood (ML) phylogenetic tree was constructed in MEGA 11 using the Jones-Taylor-Thornton (JTT) model with uniform rates among sites and 1,000 bootstrap replicates. The ML tree search employed the Nearest-Neighbor-Interchange (NNI) heuristic method, with the initial tree generated automatically by NJ/BioNJ. The resulting tree was visualized and annotated using the Interactive Tree of Life (iTOL, https://itol.embl.de/).

### 2.5 Gene structure and conserved motif analysis

Genomic sequences and coding sequences (CDS) of the *BcSnRK2* genes were extracted from the *Bombax ceiba* genome annotation file. Exon–intron structures were directly generated using TBtools (version v2.485) based on the gene annotation file. Conserved motifs of BcSnRK2 proteins were identified using the Multiple Expectation Maximization for Motif Elicitation (MEME) suite (version 5.5.9, https://meme-suite.org/meme/) with the maximum number of motifs set to 10. Functional domains were annotated using the NCBI Conserved Domain Database (CDD) (Luo et al., 2024).

### 2.6 Promoter cis-acting element analysis

The 2,000 bp upstream sequences from the start codon (ATG) of each BcSnRK2 gene were extracted from the *Bombax ceiba* genome. These promoter sequences were submitted to the PlantCARE database (https://bioinformatics.psb.ugent.be/webtools/plantcare/html/) to identify cis-acting regulatory elements. The results were visualized using the Biosequence Structure Illustrator function of TBtools 2.485.

### 2.7 Chromosomal localization and synteny analysis

The chromosomal localization of *BcSnRK2* genes in *Bombax ceiba* was analyzed using TBtools software (Version 2.485). Chromosome length data were obtained using the “Fasta Stats” function, while gene IDs and their corresponding positional information were extracted from the genome annotation file (GFF3) using the “GFF3 Gene Position Parse” and” Text Block Extract and Filter” tools. Gene density information was collected using the “Gene Density Profile” module. The distribution of *BcSnRK2* genes across chromosomes was subsequently visualized using the “Gene Location Visualize” function, providing a comprehensive view of their physical positions within the *Bombax ceiba* genome. Duplication events involving *BcSnRK2* genes were analyzed using the “MCScanX” module of TBtools, and the results were visualized with the “Advanced Circos” module. Based on the chromosomal localization information in the *Bombax ceiba* genomic GFF annotation, *BcSnRK2* genes were mapped to the 48 chromosomes. Genes were arranged in ascending order of their physical positions, from the short-arm telomere to the long-arm telomere. Genomic sequence and annotation files for *Arabidopsis thaliana* and *Populus euphratica* were downloaded from the NCBI (https://www.ncbi.nlm.nih.gov/). Syntenic blocks were identified and visualized using the “Dual Synteny Plot” module in TBtools.

### 2.8 Protein secondary and three-dimensional structure prediction

The Network Protein Sequence Analysis (NPS@) server (https://npsa.lyon.inserm.fr) was used to predict the secondary structures of the BcSnRK2 protein family. Additionally, to investigate the spatial configuration of these proteins, three-dimensional (3D) structures of BcSnRK2 proteins were generated using the SWISS-MODEL server (https://swissmodel.expasy.org/).

### 2.9 Subcellular localization assay

The subcellular localization of the identified BcSnRK2 proteins was predicted using the DeepLoc-2.1 algorithm (https://services.healthtech.dtu.dk/services/DeepLoc-2.1/). For experimental subcellular localization analysis, full-length coding sequences (including stop codons) of the nine *BcSnRK2* genes were amplified from *Bombax ceiba* cDNA using gene-specific primers (**Supplementary Table 1**). According to the instructions of the Gateway™ kit, the PCR products were cloned into the pDNOR221 vector using Gateway™ BP Clonase™ II Enzyme mix (Kit No. 11789100, Invitrogen). Subsequently, the BcSnRK2 CDSs were subcloned into pCambia1300 using Gateway™ LR Clonase™ II Enzyme mix (Kit No. 11789100, Invitrogen), resulting in in-frame fusions with the enhanced green fluorescent protein (eGFP) under the control of the constitutive *UBQ10* promoter. The resulting recombinant constructs (*UBQ10::eGFP-BcSnRK2.1–2.9*) were separately transformed into *Agrobacterium tumefaciens* strain GV3101.

*Nicotiana benthamiana* plants were grown in a growth chamber for 4–5 weeks under a 16 h light/8 h dark photoperiod at 24 °C. For transient transformation, agrobacterial cells were harvested, resuspended in infiltration buffer (10 mM MgCl_2_, 10 mM MES pH 5.6, and 150 µM acetosyringone), and adjusted to an optical density at 600 nm (OD_600_) of 0.5. The suspensions were infiltrated into the abaxial side of fully expanded leaves using a needleless syringe. At 48 h post-infiltration, leaf discs were excised and examined for eGFP fluorescence using a CLSM610 confocal laser scanning microscope (SOPTOP, China) with excitation at 488 nm and emission at 500–530 nm.

### 2.10 Protein–protein interaction prediction

To predict potential interacting partners of BcSnRK2 proteins, the amino acid sequences of the nine BcSnRK2s were submitted to the STRING database (version 12.0, https://string-db.org/) for orthology-based interaction prediction. Because the B*ombax ceiba* genome is incompletely annotated in STRING, the closest homologs of each BcSnRK2 in *Arabidopsis thaliana* were identified using reciprocal BLASTP. Specifically, BcSnRK2.2, BcSnRK2.4, BcSnRK2.5, and BcSnRK2.9 matched SRK2E (AtSnRK2.6); BcSnRK2.1, BcSnRK2.3, and BcSnRK2.6 corresponded to SRK2A (AtSnRK2.4); and BcSnRK2.7 and BcSnRK2.8 corresponded to SRK2C (AtSnRK2.8). The analysis was conducted with default settings, including an interaction confidence score threshold, to visualize predicted interactions with known abscisic acid (ABA) signaling components and potential regulatory partners.

### 2.11 Statistical analysis

All statistical analyses were performed using Origin version 2019 (OriginLab) and SPSS Statistics 22 (IBM, SPSS Statistics version 22, IBM Corporation, New York, USA). For qRT-PCR data, one-way analysis of variance (ANOVA) followed by Tukey’s honest significant difference (HSD) *post hoc* test was used to determine statistically significant differences in gene expression across different time points or tissues. P-value < 0.05 was considered statistically significant. All data are presented as the mean ± standard error (SE) or standard deviation (SD) from three biological replicates.

## 3 Results

### 3.1 Identification of SnRK2 family members in *Bombax ceiba* and analysis of phylogenetic relationship

**Figure 1:**
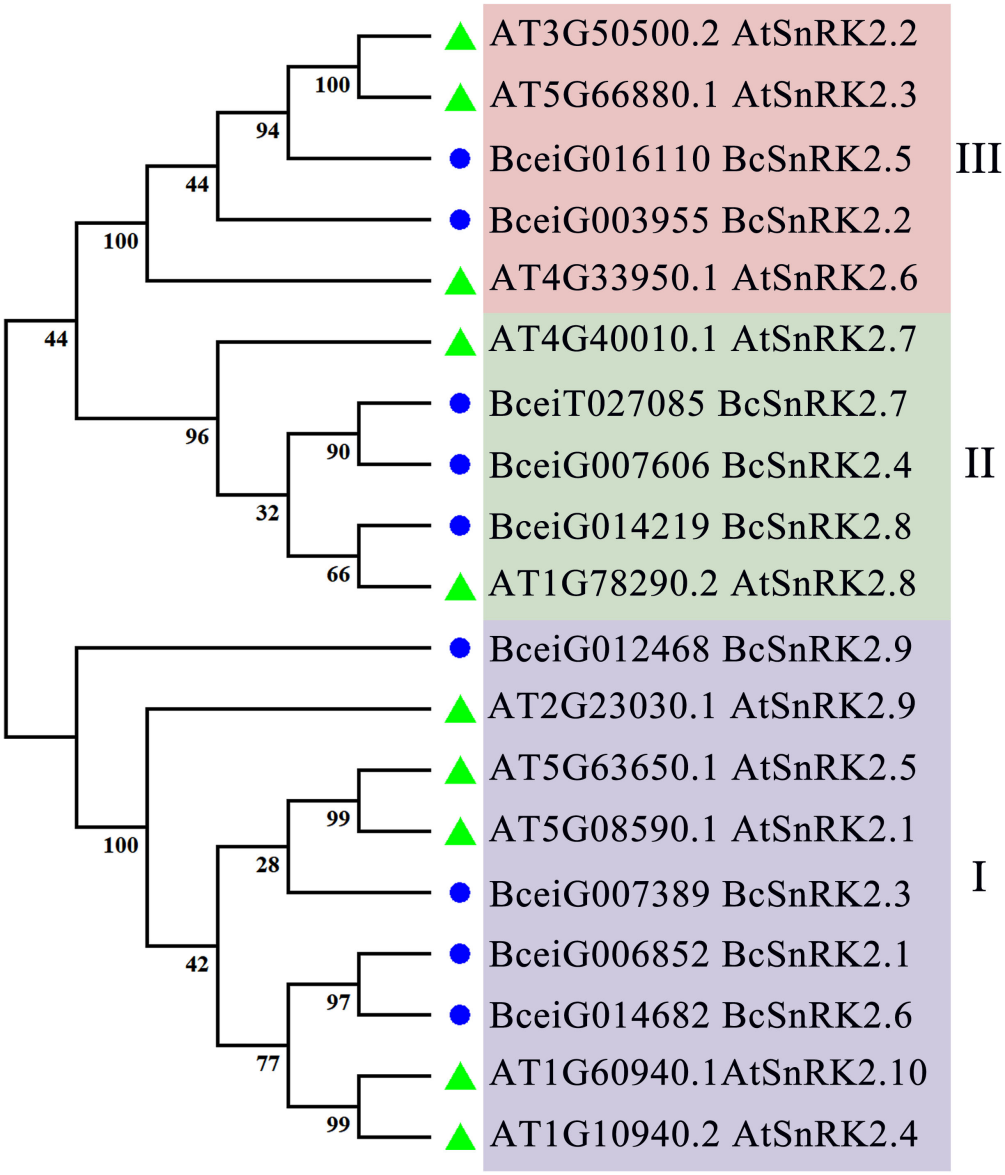
Phylogenetic relationship of SnRK2 proteins between *Bombax ceiba* and *Arabidopsis thaliana*. The maximum likelihood (ML) tree was constructed based on the full-length amino acid sequences of SnRK2 members from *Bombax ceiba* (BcSnRK2.1–BcSnRK2.9) and *Arabidopsis thaliana* (AtSnRK2.1–AtSnRK2.10). The three major clades are designated as Groups I, II, and III. Bootstrap values from 1,000 replicates are indicated at the nodes.

A total of nine SnRK2 family members were identified in *Bombax ceiba*, designated as BcSnRK2.1 to BcSnRK2.9 based on their sequence IDs (**Supplementary Table 2**). The detailed physicochemical properties of the identified BcSnRK2 proteins, including molecular weight, isoelectric point (pI), instability index, aliphatic index, and grand average of hydropathicity (GRAVY). The number of amino acid residues ranged from 308 (BcSnRK2.3) to 363 (BcSnRK2.2), with corresponding molecular weights varying between 35,519.84 Da and 41,257.75 Da. The theoretical isoelectric points (pI) exhibited considerable variation, from 4.78 (BcSnRK2.5) to 8.42 (BcSnRK2.3), indicating the presence of both acidic and basic isoforms. According to the instability index (threshold >40 for instability), only BcSnRK2.4, BcSnRK2.5, BcSnRK2.7, BcSnRK2.8, and BcSnRK2.9 are predicted to be stable, while BcSnRK2.1, BcSnRK2.2, BcSnRK2.3, and BcSnRK2.6 are predicted to be unstable. The aliphatic index ranged from 79.77 (BcSnRK2.1) to 89.01 (BcSnRK2.4), indicating a high content of aliphatic amino acids. All nine proteins displayed negative grand average of hydropathicity (GRAVY) scores, ranging from -0.450 (BcSnRK2.2) to -0.271 (BcSnRK2.8), indicating their hydrophilic nature (**Supplementary Table 3**). To further investigate the evolutionary relationships between the nine BcSnRK2 proteins from *Bombax ceiba* and the SnRK2 family members in *Arabidopsis thaliana*, we constructed a phylogenetic tree using the maximum likelihood method (**Figure 1**). The analysis showed that the SnRK2 proteins from *Bombax ceiba* and *Arabidopsis thaliana* clustered into three distinct groups (I, II, and III). Specifically, BcSnRK2.2 and BcSnRK2.5 grouped with AtSnRK2.2, AtSnRK2.3, and AtSnRK2.6 (group I); BcSnRK2.4, BcSnRK2.7, and BcSnRK2.8 clustered with AtSnRK2.7 and AtSnRK2.8 (group II); whereas BcSnRK2.1, BcSnRK2.3, and BcSnRK2.6 grouped with other AtSnRK2 members, including AtSnRK2.1, AtSnRK2.4, AtSnRK2.5, AtSnRK2.9, and AtSnRK2.10 (group III). These results suggest both functional divergence and conservation among these family members.

### 3.2 Analysis of gene and protein structure of the BcSnRK2s

**Figure 2:**
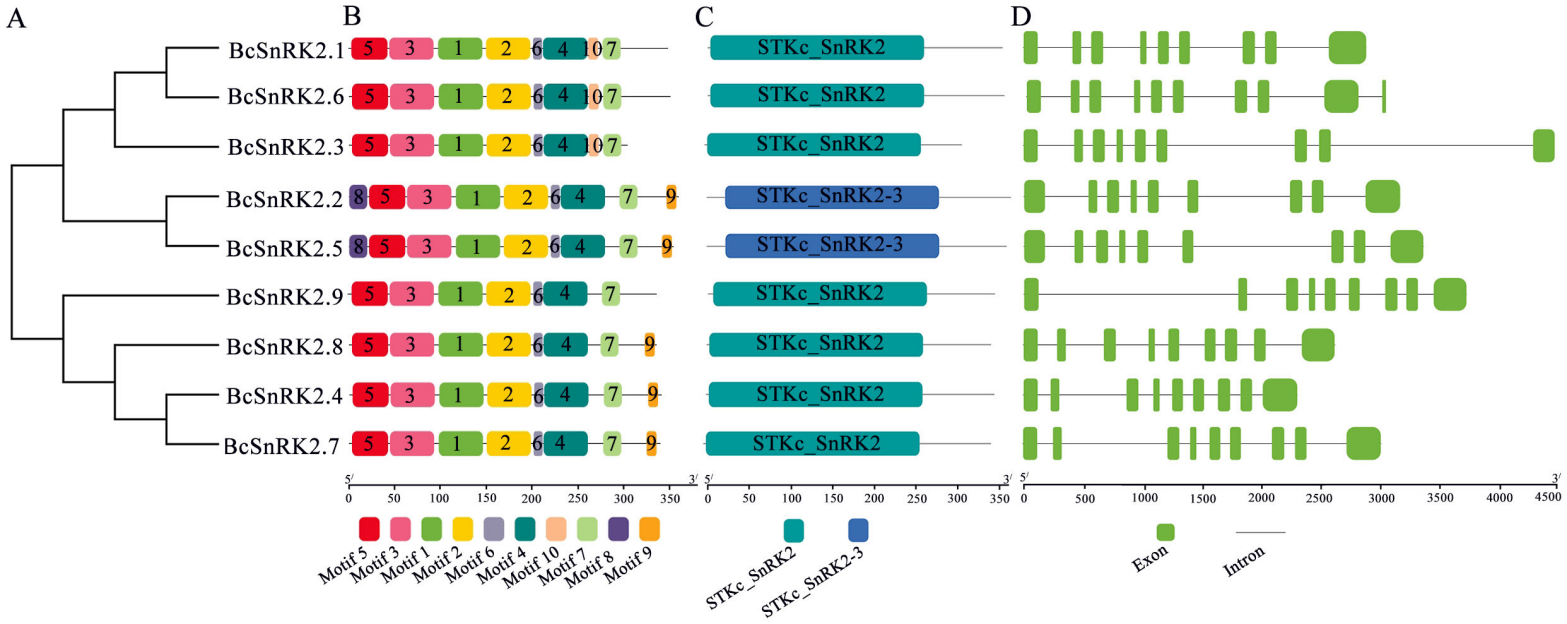
Phylogenetic relationships, conserved motifs, functional domains, and exon–intron structures of the *BcSnRK2* gene family. **(A)** Phylogenetic tree of the nine BcSnRK2 proteins. **(B)** Conserved motif distributions in BcSnRK2 proteins. Ten distinct motifs (Motif 1–10) are represented by different colored boxes. **(C)** Functional domain architecture of representative BcSnRK2 proteins. Seven of the nine BcSnRK2 members contain the STKc_SnRK2 domain (teal), while the other two harbor the STKc_SnRK2–3 domain (dark blue). **(D)** Exon–intron structures of the *BcSnRK2* genes. Green boxes represent exons, and gray lines represent introns.

To characterize the structural features of *BcSnRK2* genes in *Bombax ceiba*, we systematically analyzed their conserved protein motifs, functional domains, and exon-intron architectures. The nine identified BcSnRK2 members were grouped into three distinct clades based on phylogenetic relationships (**Figure 2A**). Conserved motif analysis using MEME identified 10 distinct motifs (designated Motifs 1–10) among the BcSnRK2 proteins. A core set of motifs (Motifs 1–7) is shared by all members. Notably, BcSnRK2.2 and BcSnRK2.5 contain two additional motifs (Motifs 8 and 9), whereas Motif 10 is exclusive to BcSnRK2.1, BcSnRK2.3, and BcSnRK2.6 (**Figure 2B**). The high conservation of motif distribution across different evolutionary branches strongly supports the phylogenetic classification. Functional domain analysis showed that the canonical STKc_SnRK2 domain, a serine/threonine kinase domain characteristic of the SnRK2 family, is present in all nine BcSnRK2 members. Notably, the domains of BcSnRK2.2 and BcSnRK2.5 are classified as STKc_SnRK2-3 (**Figure 2C**), suggesting that these two members may have evolved functional roles distinct from those of the other family members. Exon–intron structure analysis revealed that all nine *BcSnRK2* genes (*BcSnRK2.1–BcSnRK2.9*) contain nine exons each, with the exception of BcSnRK2.6, which has ten exons. (**Figure 2D**). These findings demonstrate that the BcSnRK2 family is structurally conserved across its members, particularly in functional domains, yet exhibits subfamily-specific variations in motif distribution and gene architecture, reflecting their evolutionary divergence.

### 3.3 Analysis of cis-acting elements in the promoter regions of *BcSnRK2s*

**Figure 3:**
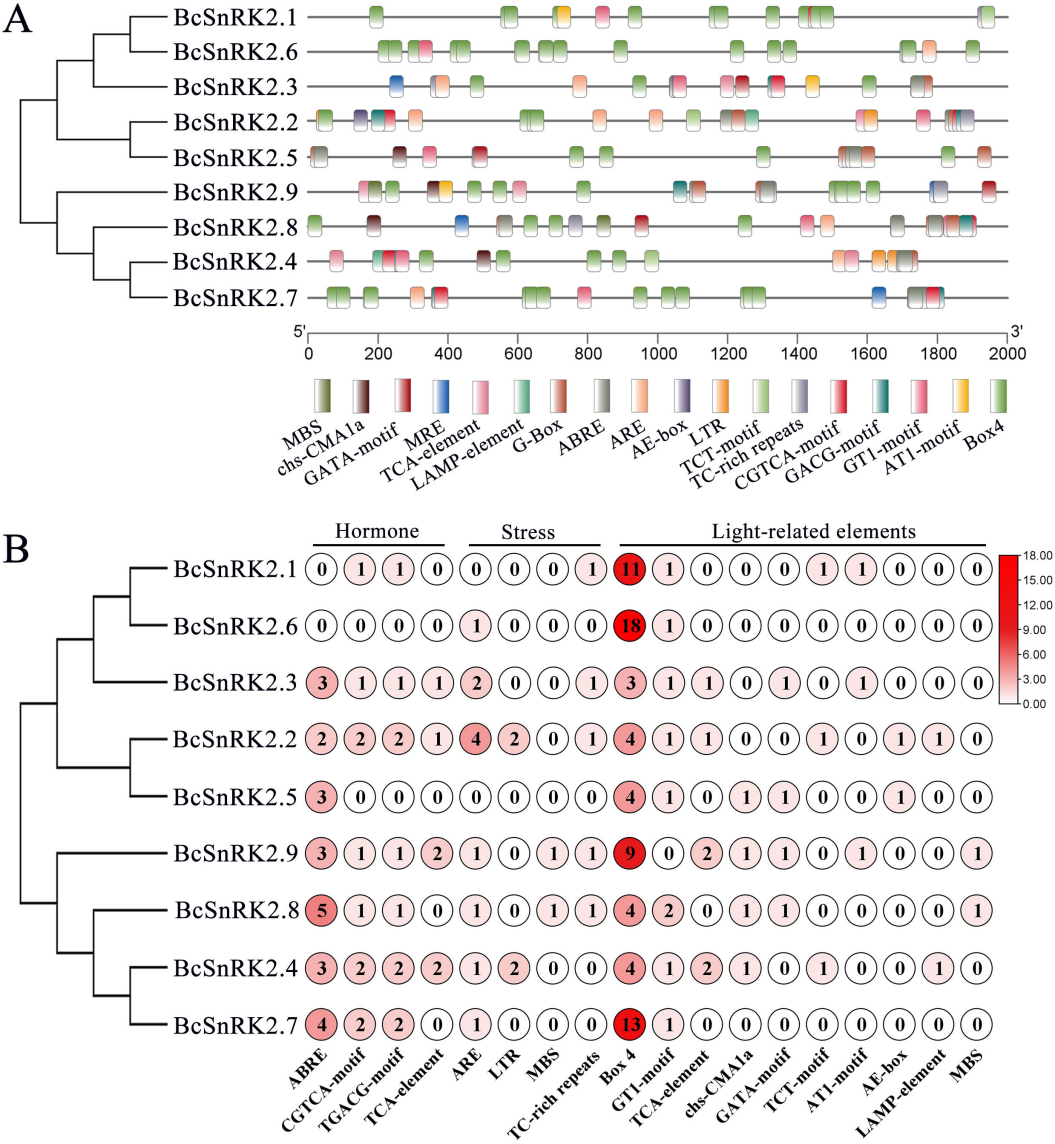
Predicted cis-elements in the promoters of *BcSnRK2* genes. **(A)** Promoter sequences (-2000 bp upstream of the start codon) of the nine *BcSnRK2* genes were analyzed using PlantCARE. Different cis-elements are represented by different colors. **(B)** Heatmap and numerical summary of predicted cis-element counts within the promoter regions (−2000 bp) of nine *BcSnRK2* genes. The color gradient from white to red represents increasing element frequency, and precise counts are displayed in each cell.

To clarify the potential transcriptional regulation and functional divergence of *BcSnRK2* genes, the 2000 bp upstream promoter sequences of nine *BcSnRK2* members were systematically identified and annotated for cis-acting regulatory elements (**Figure 3A**). A total of 18 types of cis-acting elements were identified and classified into three major categories: hormone-responsive, stress-responsive, and light-related elements (**Figure 3B**). Among the hormone-responsive elements, ABRE, CGTCA-motif/TGACG-motif, and TCA-element were widely distributed in the promoters of *BcSnRK2* genes. The ABRE element, which mediates ABA responsiveness, was detected in almost all *BcSnRK2* genes (except BcSnRK2.1 and BcSnRK2.6), with BcSnRK2.7 and *BcSnRK2.8* containing the highest copy numbers (4 and 5, respectively). This suggests that *BcSnRK2* family genes are primarily involved in ABA-mediated signaling pathways. The MeJA-responsive CGTCA- and TGACG-motifs were simultaneously enriched in *BcSnRK2.1*, *BcSnRK2.2*, *BcSnRK2.3*, *BcSnRK2.4*, *BcSnRK2.7*, *BcSnRK2.8*, and *BcSnRK2.9*, indicating that these members may participate in methyl jasmonate signal transduction. Additionally, the salicylic acid-responsive TCA-element was identified in *BcSnRK2.2*, *BcSnRK2.3*, *BcSnRK2.4*, and *BcSnRK2.9* (**Figure 3B**), suggesting that *BcSnRK2* genes may integrate multiple phytohormone signals to regulate plant growth and stress adaptation. Regarding stress-responsive cis-elements, LTR, TC-rich repeats, MBS, and ARE were identified. The low-temperature-responsive LTR element was found in *BcSnRK2.2* and *BcSnRK2.4*; defense- and stress-related TC-rich repeats were present in *BcSnRK2.1*, *BcSnRK2.2*, *BcSnRK2.3*, *BcSnRK2.8*, and *BcSnRK2.9*; the drought-inducible MBS element was detected in *BcSnRK2.8* and *BcSnRK2.9*; and the anaerobic induction-related ARE element was widely distributed across *BcSnRK2.2*, *BcSnRK2.3*, *BcSnRK2.4*, *BcSnRK2.6*, *BcSnRK2.7*, *BcSnRK2.8*, and *BcSnRK2.9* (**Figure 3B**). These results indicate that different *BcSnRK2* members have acquired distinct cis-element combinations to cope with low temperature, drought, anaerobic stress, and pathogen defense. Light-associated elements, including Box 4, GT1-motif, GATA-motif, chs-CMA1a, TCT-motif, AT1-motif, AE-box, and LAMP-element, were also prevalent in *BcSnRK2* promoter regions. Notably, Box 4, which is part of a conserved DNA module involved in light responsiveness, was the most abundant element, with particularly high copy numbers in *BcSnRK2.6* (18 copies) and *BcSnRK2.7* (13 copies). Multiple copies of these light-responsive elements were widely distributed across all *BcSnRK2* genes, indicating that the expression of the *BcSnRK2* family is tightly regulated by light signals.

Overall, the promoters of BcSnRK2 genes contain abundant and diverse cis-acting elements associated with phytohormone signaling, abiotic stress tolerance, and light response. The distinct distribution patterns of these elements among different BcSnRK2 members provide essential evidence for their functional differentiation and their roles in integrating multiple signals within regulatory networks.

### 3.4 Chromosomal location and synteny analysis of *BcSnRK2* genes

**Figure 4:**
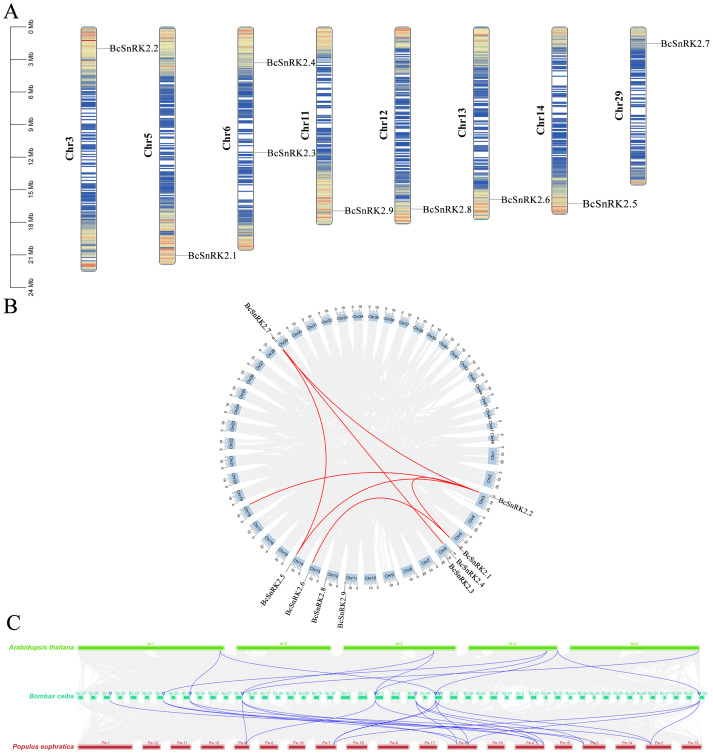
Genomic distribution and synteny analysis of *BcSnRK2* genes. **(A)** Schematic representation of the chromosomal distribution of *Bombax ceiba BcSnRK2* genes. **(B)** Chromosomal distribution and collinearity of *BcSnRK2* genes. Chromosomes are depicted as vertical ideograms with scale bars; gene positions are indicated outside the chromosomes. Dark curves denote segmental duplication pairs. **(C)** Interspecific synteny of *BcSnRK2* genes among *Bombax ceiba*, *Arabidopsis thaliana*, and *Populus euphratica*. Chromosomes are shown as horizontal columns. The gray curves in the circular diagram represent segmental duplications across the entire *Bombax ceiba* genome, while the highlighted curves denote segmental duplications involving *BcSnRK2* genes.

According to the gene loci information, the nine *BcSnRK2* genes are distributed across eight chromosomes. Specifically, *BcSnRK2.1*, *BcSnRK2.2*, and *BcSnRK2.5* are located on chromosomes Chr5, Chr3, and Chr14, respectively. Chromosome Chr6 contains two members, *BcSnRK2.3* and *BcSnRK2.4*. The remaining genes, including *BcSnRK2.6*, *BcSnRK2.7*, *BcSnRK2.8*, and *BcSnRK2.9* are situated on chromosomes Chr13, Chr29, Chr12, and Chr11, respectively (**Figure 4A**). Collinear genes refer to homologous genes that arise from genome duplication and maintain consistent gene order across different genomes, paralogous genes refer to homologous genes that arise from gene duplication events after speciation (Cannon et al., 2004). Collinearity analysis of the BcSnRK2 gene family revealed a total of seven gene pairs among the nine *BcSnRK2* genes, four of which were located on chromosome 3 (**Figure 4B**). The presence of collinearity between genes of different species often indicates functional similarity. Therefore, we performed a synteny analysis of the BcSnRK2 gene families in *Bombax ceiba*, *Arabidopsis thaliana*, and *Populus euphratica*. A total of 9 syntenic gene pairs were identified between *Arabidopsis* and *Bombax ceiba*, whereas 19 syntenic gene pairs were found between *Bombax ceiba* and *Populus euphratica* (**Figure 4C**). This suggests that segment duplications may be the main expansion mode of the *BcSnRK2* gene family.

### 3.5 Analysis of the secondary structure and three-dimensional structure of the BcSnRK2 protein

**Figure 5:**
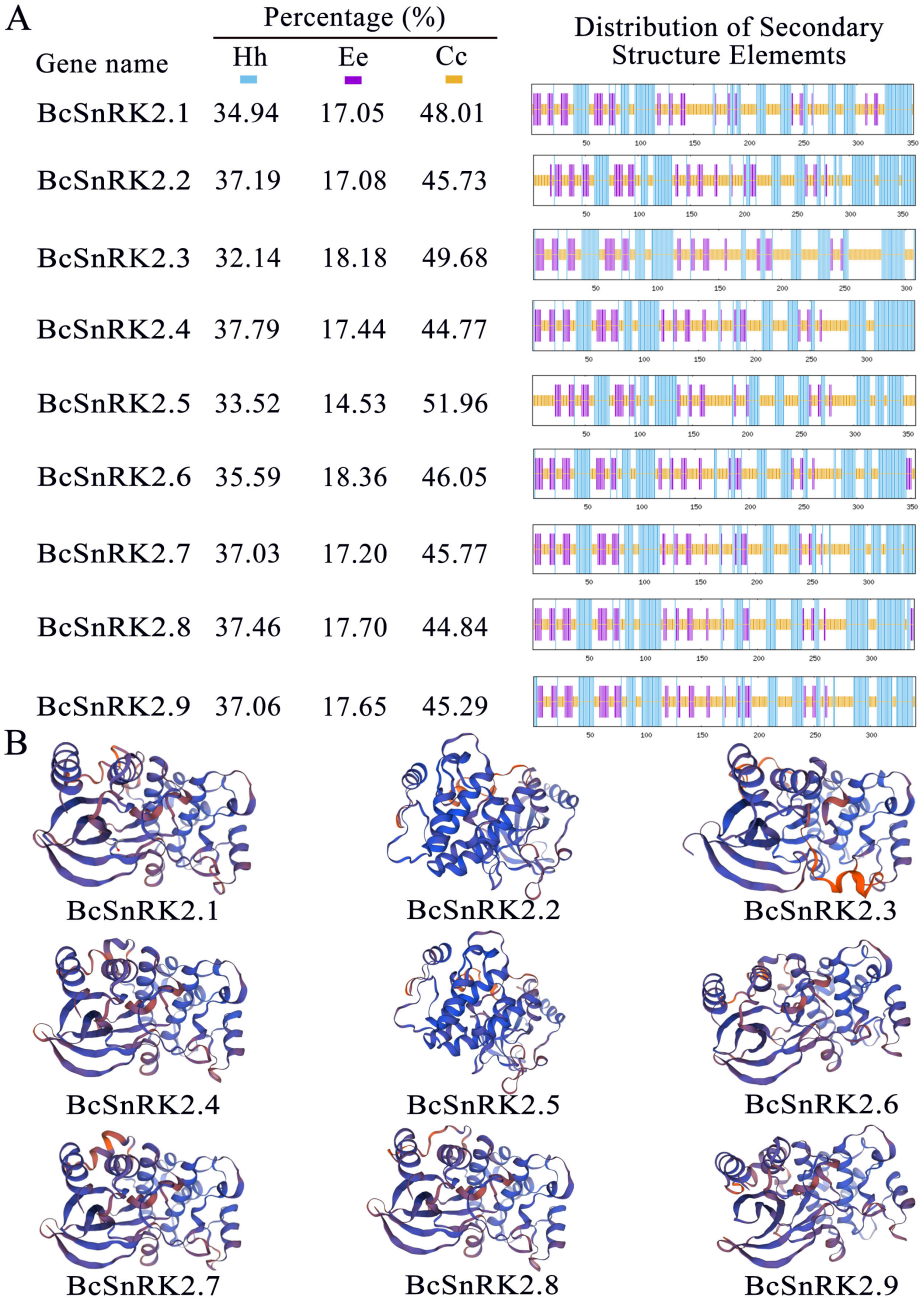
Secondary structure and 3D structure prediction of BcSnRK2 proteins. **(A)** Secondary structure prediction of BcSnRK2 proteins. Hh (alpha helix), Ee (extended strand), and Cc (random coil). **(B)** Protein 3D structure prediction models of the *BcSnRK2* gene family.

The secondary structure composition of all BcSnRK2 proteins includes α-helices (Hh), extended strands (Ee), and random coils (Cc). Among these, random coils are the most abundant structural type across all analyzed proteins, with proportions ranging from 44.77% to 51.96%. α-Helices range from 32.14% to 37.79%, while extended strands account for 14.53% to 18.36% (**Figure 5A**). The high proportions of α-helices and random coils suggest that these two elements constitute the predominant conformational framework of the BcSnRK2 proteins. Random coils may contribute to protein flexibility by facilitating conformational changes and connecting more rigid α-helices and extended strands, likely stabilized by hydrogen bonding interactions. In contrast, α-helices, stabilized by regular intra-chain hydrogen bonds, are important for protein stability and may participate in domain formation and molecular interactions. Furthermore, the predicted three-dimensional (3D) structures of the BcSnRK2 proteins reveal that BcSnRK2.2 and BcSnRK2.5 share highly conserved tertiary structures, while the tertiary structures of the remaining seven BcSnRK2s are also highly conserved (**Figure 5B**). Despite minor variations in loop regions and the orientation of some secondary structural elements, the overall topology and spatial arrangement of functional domains remain largely consistent across the nine paralogs, indicating strong structural conservation within the BcSnRK2 family.

### 3.6 Tissue-specific expression profiles of *BcSnRK2s* genes

**Figure 6:**
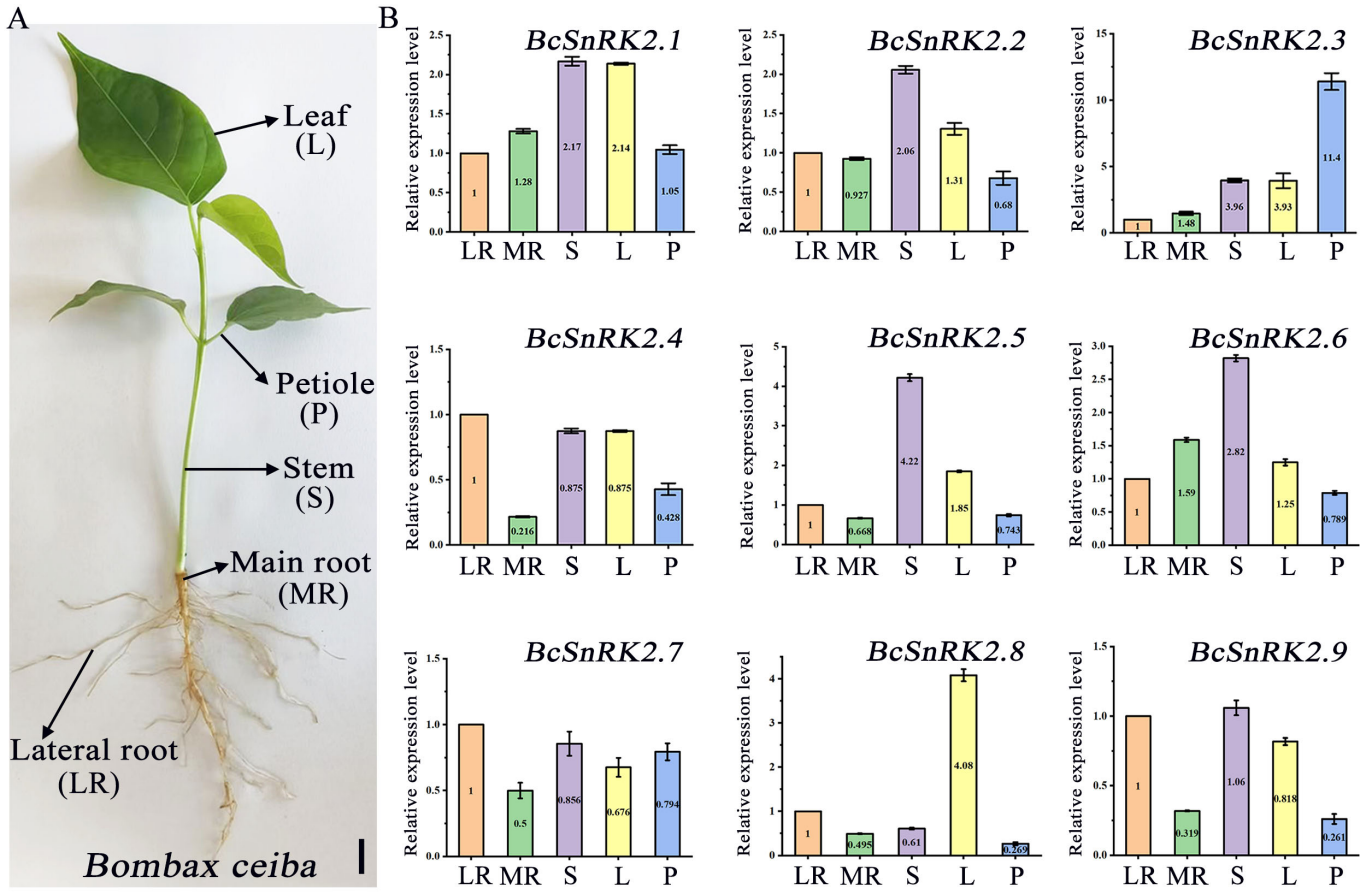
Tissue-specific expression profiles of *BcSnRK2* genes in *Bombax ceiba*. **(A)** Schematic diagram of tissue sampling locations: lateral roots (LR), main roots (MR), stems (S), leaves (L), and petioles (P). Scale bars = 1 cm. **(B)** The relative expression levels of *BcSnRK2.1–9* in different tissues were measured by qRT-PCR, with the expression level in lateral roots (LR) set to 1.00. Expression levels were normalized to the internal control gene *BcUBQ5*, and values represent the means ± standard deviation (SD) of three biological replicates.

To investigate the potential physiological roles of BcSnRK2 genes in different tissues of *Bombax ceiba*, we examined their relative expression levels in lateral roots (LR), main roots (MR), stems (S), leaves (L), and petioles (P) using qRT-PCR (**Figure 6A**). The nine BcSnRK2 genes displayed distinct tissue-preferential expression patterns (**Figure 6B**). Notably, *BcSnRK2.3* was expressed at extremely high levels in petioles (mean = 11.40), suggesting a specialized role in vascular or mechanical tissues. *BcSnRK2.5* and *BcSnRK2.6* were predominantly expressed in stems (means = 4.20 and 2.82, respectively), whereas *BcSnRK2.8* showed a striking leaf-specific pattern (mean = 4.08), indicating potential involvement in photosynthesis or leaf stress responses. In contrast, *BcSnRK2.1* exhibited relatively uniform expression across all tissues (ranging from 1.05 to 2.17), implying a constitutive or housekeeping function. *BcSnRK2.2* and *BcSnRK2.7* displayed moderate expression without strong tissue preference, while *BcSnRK2.4* and *BcSnRK2.9* were consistently expressed at very low levels across all tissues examined. Collectively, these tissue-specific expression profiles reveal substantial functional diversification within the BcSnRK2 gene family, with certain members likely performing specialized roles in specific tissues and others serving more general regulatory functions in *Bombax ceiba*.

### 3.7 Subcellular localization of BcSnRK2s

**Figure 7:**
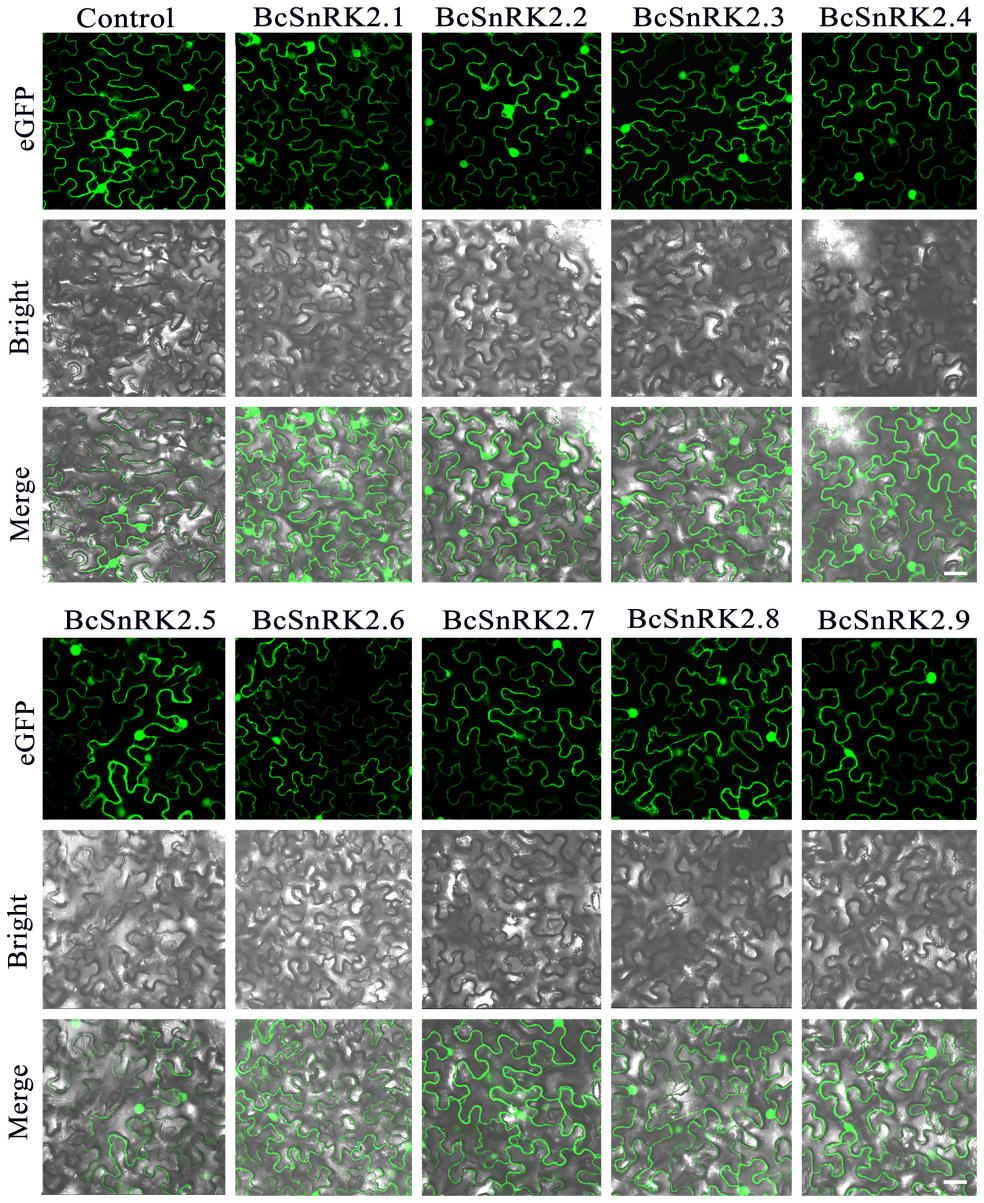
Subcellular localization of BcSnRK2.1–2.9 proteins in *Nicotiana benthamiana* leaves. The nine recombinant constructs (*UBQ10::eGFP-BcSnRK2.1–2.9*) were transiently expressed in *Nicotiana benthamiana* leaves via *Agrobacterium*-mediated transformation, and eGFP fluorescence signals were observed under a confocal microscope. For each construct, images show eGFP fluorescence (green), bright-field (Bright), and merged channels. Control: *UBQ10::eGFP*. Scale bars = 25 μm.

To investigate the potential subcellular distribution of the BcSnRK2 family members, subcellular localization predictions were performed using DeepLoc-2.1. The analysis revealed that all nine BcSnRK2 proteins (BcSnRK2.1–2.9) showed the highest prediction scores for the cytoplasm, with values ranging from 0.65 to 0.77. The nucleus was predicted as the second most probable compartment, with scores between 0.55 and 0.67 (**Supplementary Figure 2**). To further validate the subcellular localization of BcSnRK2 proteins, we constructed nine recombinant plasmids, each containing the coding sequence of a BcSnRK2 gene fused with enhanced green fluorescent protein (eGFP) under the control of the UBQ10 promoter (*UBQ10::eGFP-BcSnRK2.1–2.9*). A control plasmid expressing free eGFP driven by the same promoter (*UBQ10::eGFP*) was also constructed. These plasmids were delivered into *Nicotiana benthamiana* leaves via *Agrobacterium*-mediated transient transformation. The results showed that, compared with the uniform distribution of GFP fluorescence in the cytoplasm and nucleus observed in the free eGFP control, all nine identified eGFP-BcSnRK2 fusion proteins were localized to the plasma membrane (PM). In addition, each fusion protein displayed diffuse cytoplasmic signals and clear nuclear accumulation (**Figure 7**). Overall, these results indicate that BcSnRK2.1 to BcSnRK2.9 are primarily distributed at the PM, in the cytoplasm, and in the nucleus, a finding consistent with previous studies (Liu et al., 2024).

### 3.8 Expression patterns of *BcSnRK2* gene family members in response to 10% PEG6000-simulated drought stress

**Figure 8:**
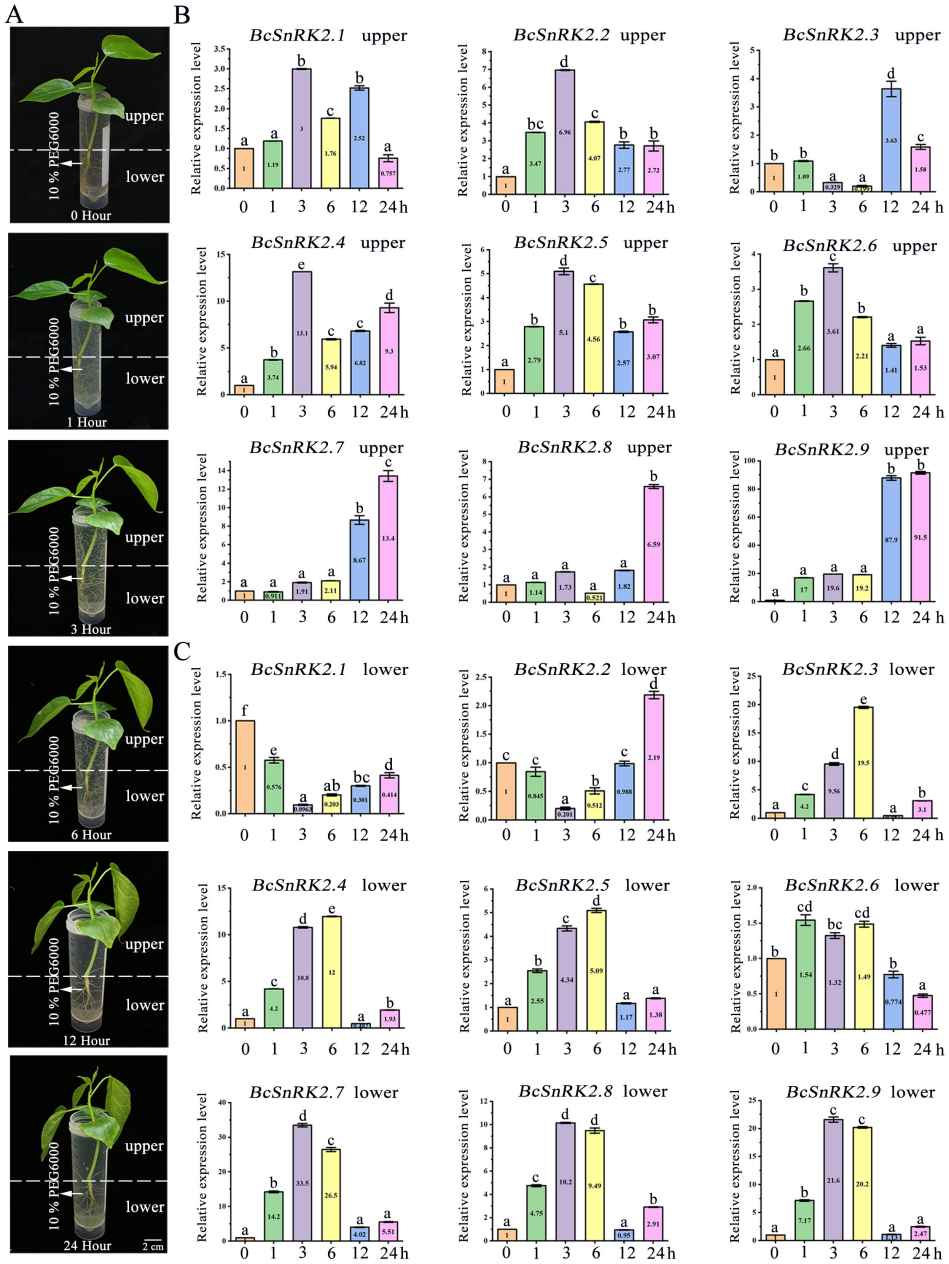
The expression levels of the *BcSnRK2* gene family in the upper and lower regions of *Bombax ceiba* seedlings subjected to drought stress simulated by 10% PEG6000 were assessed using qRT-PCR. **(A)** Phenotypes of seedlings over a 24-hour time course following treatment with 10% PEG6000. The dashed line indicates the boundary between the upper and lower tissues harvested for gene expression analysis. Scale bar = 2 cm. **(B)** Relative expression levels of *BcSnRK2.1* to *BcSnRK2.9* in the upper tissues. **(C)** Relative expression levels of *BcSnRK2.1* to *BcSnRK2.9* in the lower tissues. *BcUBQ5* was used as the reference gene. Data are presented as the mean ± standard error (SE) from three biological replicates. Different lowercase letters above the bars denote statistically significant differences (p < 0.05), as determined by one-way ANOVA followed by Tukey’s multiple range test.

To characterize the transcriptional responses of *BcSnRK2* family genes to drought stress, seedlings were treated with 10% PEG6000 over a 24-hour period. Relative expression levels of nine *BcSnRK2* genes were measured in two tissue types (upper and lower parts) at six time points: 0, 1, 3, 6, 12, and 24 hours post-treatment (**Figure 8A**). Significant temporal and tissue-specific expression patterns were observed for all tested genes. In the upper tissues, *BcSnRK2.9* showed rapid and sustained up-regulation, with expression levels rising sharply from 1 h (17.0) to 12 h (87.9) and remaining high at 24 h (91.53). *BcSnRK2.7* was progressively induced and peaked at 24 h. BcSnRK2.8 displayed a moderate but fluctuating increase, reaching 6.59 at 24 h. Notably, *BcSnRK2.5* and *BcSnRK2.3* were transiently induced at early time points (1–6 h): BcSnRK2.5 peaked at 3 h (5.1) and *BcSnRK2.3* at 12 h (3.63), before declining. In contrast, *BcSnRK2.2* and *BcSnRK2.4* showed gradual induction, while *BcSnRK2.1* and *BcSnRK2.6* remained relatively low throughout the time course, with BcSnRK2.6 decreasing slightly after 6 h (**Figure 8B**).

In the lower tissues, a markedly different expression profile was observed. *BcSnRK2.7* exhibited the most dramatic induction, rising to 33.5 at 3 h and remaining elevated (26.5 at 6 h, 5.51 at 24 h). BcSnRK2.3 also displayed a strong and sustained response, peaking at 6 h (19.5). *BcSnRK2.9* was moderately induced, with a peak at 3 h (21.6) followed by a sharp decline to near baseline at 12 h and 24 h; *BcSnRK2.8* followed a similar but weaker pattern. Interestingly, *BcSnRK2.4* and *BcSnRK2.5* were moderately induced (up to 12.0 and 5.09, respectively), whereas *BcSnRK2.2* showed a delayed increase (2.19 at 24 h). *BcSnRK2.1* and *BcSnRK2.6* remained largely unchanged or were slightly suppressed throughout the treatment (**Figure 8C**). Collectively, these results demonstrate that *BcSnRK2* family members respond to PEG-induced drought stress in a tissue- and time-dependent manner. Notably, *BcSnRK2.9* and *BcSnRK2.7* showed strong and sustained activation in upper tissues, suggesting roles in shoot responses to water deficit, whereas *BcSnRK2.7* and *BcSnRK2.3* were highly responsive in lower tissues, implicating them in root or basal tissue adaptation. The contrasting expression patterns between upper and lower tissues highlight the functional divergence of *BcSnRK2* genes during drought acclimation. These results indicate that individual *BcSnRK2* family members may function in distinct spatial and temporal contexts during plant adaptation to drought stress.

### 3.9 Predicting protein–protein interactions of the BcSnRK2s

**Figure 9:**
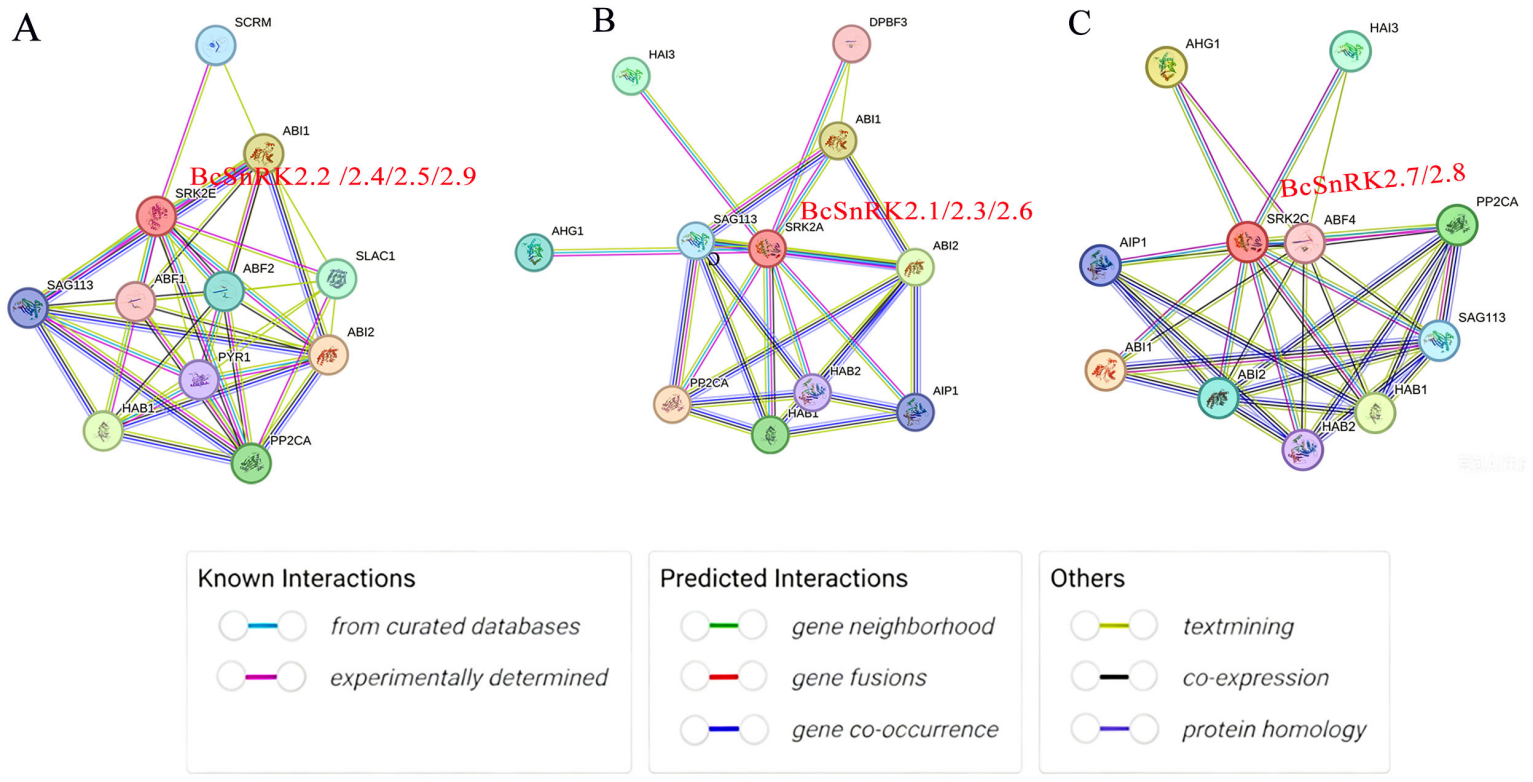
STRING analysis of nine BcSnRK2 proteins. **(A)** Protein–protein interaction network of BcSnRK2.2, BcSnRK2.4, BcSnRK2.5, and BcSnRK2.9. **(B)** Protein–protein interaction network of BcSnRK2.1, BcSnRK2.3, and BcSnRK2.6. **(C)** Protein–protein interaction network of BcSnRK2.7 and BcSnRK2.8.

To further explore the biological functions and regulatory networks of the BcSnRK2 proteins, protein-protein interaction (PPI) networks were constructed using the STRING database (http://string-db.org, accessed on 4 May 2026). Leveraging the high evolutionary conservation of SnRK2 kinases, the interaction profiles of BcSnRK2s were inferred based on their closest homologs in *Arabidopsis thaliana*. For the network centered on SRK2E (AtSnRK2.6, homologous to BcSnRK2.2/2.4/2.5/2.9; **Figure 9A**), the analysis revealed interactions with multiple core components of the ABA signaling pathway. These included several clade A type 2C protein phosphatases (PP2Cs) that act as key repressors of ABA signaling: ABI1, ABI2, HAB1, PP2CA, and SAG113. Notably, PP2CA is a major negative regulator of ABA responses during seed germination and cold acclimation, and prevents stomatal closure by inactivating the S−type anion efflux channel SLAC1 and its activator SRK2E. SAG113 specifically acts as a negative regulator of ABA signaling for stomatal closure and controls water loss during leaf senescence. The network also captured interactions with the ABA receptor PYR1, which is required for ABA-mediated responses such as stomatal closure and germination inhibition, and the guard cell S−type anion channel SLAC1, a critical effector of ABA-induced stomatal closure. In addition, SnRK2E interacted with the ABA-responsive transcription factors ABF1 and ABF2, which bind to ABA-responsive elements (ABREs) to regulate downstream stress responses, as well as the cold−responsive transcription factor ICE1 (SCRM), which controls the cold-induced expression of *CBF/DREB1* genes. For the network centered on SRK2A (AtSnRK2.4, homologous to BcSnRK2.1/2.3/2.6; **Figure 9B**), the interaction profile was also highly enriched in ABA signaling components, with several unique interactors. SnRK2A was predicted to interact with the core PP2C phosphatases ABI1, ABI2, HAB1, HAB2, PP2CA, and SAG113, consistent with the canonical SnRK2-PP2C regulatory module. This network further included additional PP2C family members, such as AHG1 (a negative regulator of ABA responses during seed germination), HAI3, and AIP1 (which negatively regulates the potassium channel AKT1 via dephosphorylation). Furthermore, SnRK2A interacted with the ABA-responsive transcription factor DPBF3, which binds to both embryo−specific elements and ABREs. For the network centered on SRK2C (AtSnRK2.8, homologous to BcSnRK2.7/2.8; **Figure 9C**), the analysis revealed an interaction profile highly similar to those of SRK2E and SRK2A. SRK2C was predicted to interact with the same set of core ABA signaling components, including clade A PP2Cs (ABI1, ABI2, HAB1, HAB2, PP2CA, AHG1, HAI3, AIP1, SAG113), the ABA receptor PYR1, the SLAC1 anion channel, and the transcription factors ABF1/ABF2/ABF3/ABF4, DPBF3, and ICE1. Collectively, these interaction networks demonstrate that three groups of BcSnRK2 proteins function as central nodes in ABA-dependent signaling pathways, associating with core ABA receptors, negative regulators (PP2Cs), ion channels, and downstream transcription factors involved in stress responses. These findings strongly suggest that the *BcSnRK2* genes are embedded in conserved ABA signaling modules and likely play pivotal roles in ABA-mediated stress responses, stomatal regulation, and abiotic stress adaptation in *Bombax ceiba.*

## 4 Discussion

### 4.1 A conserved SnRK2 family with nine members in *Bombax ceiba*

The SnRK2 family size varies considerably among plant species. In *Bombax ceiba*, we identified nine SnRK2 members, which is comparable to the 10 members found in *Arabidopsis thaliana* and rice, similar to the 11 members in poplar, but fewer than the 22 members in soybean (Fujii et al., 2007; Kobayashi et al., 2004; Jin et al., 2025; Zhao et al., 2017). The high-quality *Bombax ceiba* genome sequence supports the completeness of our identification (Gao et al., 2018; Yuan et al., 2025). This number is consistent with the typical SnRK2 family size observed in many angiosperms, suggesting that *Bombax ceiba* has retained a canonical SnRK2 repertoire without substantial lineage specific expansion or contraction. Notably, a genome wide investigation of two jute species (*Corchorus olitorius* and *C. capsularis*) identified only seven *SnRK2* genes (Ahmed et al., 2023), indicating that some variation in family size exists within the *Malvaceae*, though *Bombax ceiba* appears to have maintained a near complete set. All nine BcSnRK2 proteins contain the characteristic STKc_SnRK2 or related kinase domains (Kulik et al., 2011), demonstrating that the core biochemical function of SnRK2 kinases, serine/threonine phosphorylation, has been evolutionarily conserved.

Phylogenetic analysis placed BcSnRK2.2 and BcSnRK2.5 in Group I, BcSnRK2.4, BcSnRK2.7, and BcSnRK2.8 in Group II, and BcSnRK2.1, BcSnRK2.3, and BcSnRK2.6 in Group III (**Figure 1**). Group III members are typically activated by ABA and strongly implicated in drought signaling, whereas Group I and II members are generally considered ABA insensitive or weakly responsive (Yoshida et al., 2002; Krzywińska et al., 2016; Soma et al., 2020). The presence of three Group III members in *Bombax ceiba* (BcSnRK2.1, BcSnRK2.3, and BcSnRK2.6) suggests functional redundancy or subfunctionalization within the ABA dependent pathway. Notably, while the promoter regions of all *BcSnRK2* genes contain ABRE elements, their distribution varies considerably among members. *BcSnRK2.7* and *BcSnRK2.8* harbor the highest copy numbers (4 and 5, respectively), whereas *BcSnRK2.1* and *BcSnRK2.6* lack detectable ABRE elements (**Figure 3**). This variation in ABRE distribution may underlie differential ABA responsiveness among *BcSnRK2* members, as documented in *Arabidopsis* and *rice* (Yoshida et al., 2002; Kobayashi et al., 2004).

Collinearity analysis revealed seven gene pairs among the nine BcSnRK2 genes, with four pairs located on chromosome 3 (**Figure 4B**). The identification of syntenic relationships between *Bombax ceiba* and both *Arabidopsis thaliana* (9 pairs) and *Populus euphratica* (19 pairs) suggests that segmental duplications have contributed to the expansion and maintenance of the BcSnRK2 gene family. The presence of collinearity across these divergent species further indicates that the SnRK2 family has remained evolutionarily stable since the divergence of these lineages. From an evolutionary perspective, the retention of nine SnRK2 members in *Bombax ceiba*, neither substantially expanded nor reduced, suggests that this gene number may represent an optimal balance between functional diversity and regulatory efficiency for drought adaptation in tropical tree species. We emphasize, however, that while our data support gene number conservation and structural conservation, formal evolutionary analyses (e.g., estimation of nonsynonymous/synonymous substitution rates, dN/dS, to detect signatures of selection, or genotype environment association studies) would be required to establish adaptive significance. In summary, the *Bombax ceiba* SnRK2 family exemplifies how a moderately sized yet structurally conserved gene family can mediate rapid drought responses, providing a foundation for functional validation and population level investigations in a non-model woody species.

### 4.2 Tissue-specific expression patterns reflect functional diversification

The nine BcSnRK2 genes exhibited markedly distinct tissue-preferential expression patterns. *BcSnRK2.3* showed extremely high expression in petioles (11.4 relative to lateral roots), whereas *BcSnRK2.5* and *BcSnRK2.6* were predominantly expressed in stems, and *BcSnRK2.8* displayed a striking leaf-specific pattern. *BcSnRK2.1* exhibited relatively uniform expression across all tissues, suggesting a constitutive or housekeeping function (**Figure 6**). Such tissue-specific expression implies that different *BcSnRK2* paralogs have adopted specialized roles in the development or local stress responses of particular organs. For example, the high expression of *BcSnRK2.3* in petioles may be related to the regulation of vascular bundle development or hydromechanical signaling under water deficit, as petioles play a key role in water transport and leaf positioning. Similarly, the leaf-specific expression of *BcSnRK2.8* suggests potential involvement in photosynthesis or leaf stress responses, whereas stem-preferential expression of *BcSnRK2.5* and *BcSnRK2.6* may reflect roles in mechanical support or cambial activity.

This functional diversification mirrors observations in other species. In *Arabidopsis*, *SnRK2* family members also exhibit divergent expression patterns associated with distinct physiological roles. *SnRK2.6* (*OST1*) is specifically expressed in guard cells and regulates stomatal closure, whereas *SnRK2.2* and *SnRK2.3* are more broadly expressed and function redundantly in seed germination and seedling growth (Fujii et al., 2007). *SnRK2.4*, on the other hand, is involved in root growth regulation under salt stress (Krzywińska et al., 2016). These findings indicate that tissue-preferential expression is a conserved feature of SnRK2 genes across angiosperms. Thus, the nine *BcSnRK2* members in *Bombax ceiba* have likely undergone clear subfunctionalization at the transcriptional level, enabling specialized physiological roles while maintaining core stress signaling functions.

### 4.3 Dynamic drought responses and conserved ABA signaling modules of BcSnRK2s

Drought stress simulated by 10% PEG6000 induced rapid and pronounced changes in *BcSnRK2* transcript levels, with striking temporal and tissue-specific patterns (**Figure 8**). In upper tissues, *BcSnRK2.9* showed the most dramatic and sustained up-regulation, peaking at 87.9 at 12 h and remaining high at 24 h (91.5). *BcSnRK2.7* was progressively induced and peaked at 24 h, whereas *BcSnRK2.5* and *BcSnRK2.3* were transiently induced at early time points (1–6 h). In contrast, *BcSnRK2.1* and *BcSnRK2.6* remained at relatively low levels throughout the time course. In lower tissues, a markedly different expression profile was observed: *BcSnRK2.7* exhibited the most dramatic induction (33.5 at 3 h), followed by *BcSnRK2.3* (19.5 at 6 h) and *BcSnRK2.9* (21.6 at 3 h). Notably, *BcSnRK2.4* and *BcSnRK2.5* were moderately induced in lower tissues, whereas *BcSnRK2.1* and *BcSnRK2.6* remained largely unchanged.

Several findings merit particular attention. First, *BcSnRK2.9*, a Group II member not typically considered ABA activated, showed the strongest and most sustained upregulation in upper tissues. Given the presence of multiple ABRE elements (5 copies) and MeJA responsive elements in its promoter (**Figure 3**), this result suggests that certain Group II *SnRK2s* in *Bombax ceiba* may have acquired strong osmotic stress inducibility via crosstalk with ABA or jasmonate signaling. Second, *BcSnRK2.7* (Group II) was highly responsive in both upper and lower tissues but with distinct temporal dynamics: gradual induction in upper tissues (peaking at 24 h) versus rapid induction in lower tissues (peaking at 3 h), suggesting tissue specific regulatory mechanisms. Third, the contrasting expression patterns between upper and lower tissues highlight the functional divergence of *BcSnRK2* genes during drought acclimation, with certain members (e.g., *BcSnRK2.9* and *BcSnRK2.7*) playing dominant roles in shoot responses, whereas others (e.g., *BcSnRK2.7* and *BcSnRK2.3*) are more important in root or basal tissue adaptation.

Protein–protein interaction predictions using the STRING database revealed that all three groups of BcSnRK2 proteins are predicted to interact with core ABA signaling components, including the ABA receptor PYR1, clade A type 2C protein phosphatases (ABI1, ABI2, HAB1, PP2CA, SAG113), the guard cell S type anion channel SLAC1, and downstream transcription factors (ABF1, ABF2, ABF3, ABF4, DPBF3, ICE1) (**Figure 9**). This is consistent with the canonical SnRK2 PP2C regulatory module, as even Group I/II SnRK2s in *Arabidopsis* have been shown to interact with PP2Cs under certain contexts (Krzywińska et al., 2016), and ABRE elements were detected in most BcSnRK2 promoters (**Figure 3**). Therefore, we propose that in *Bombax ceiba*, the distinction between ABA dependent and ABA independent pathways may be less rigid, and all nine BcSnRK2s likely participate in an integrated ABA osmotic stress network. The predicted interaction with SLAC1 is particularly noteworthy, as it suggests a direct role in regulating stomatal closure, warranting experimental validation through Y2H, co-IP, or BiFC assays. It should be noted that the upper versus lower tissue sampling strategy used in the PEG treatment experiment was designed to capture systemic, whole plant compartment level responses rather than organ specific expression patterns. While this approach effectively revealed distinct temporal dynamics between shoot and root compartments, it does not resolve finer scale expression differences among individual organs such as leaves, stems, or specific root zones. Future studies on the key candidate genes identified here, particularly BcSnRK2.9, BcSnRK2.7, and BcSnRK2.3, would benefit from more refined spatial analysis, such as tissue dissection at the organ level or *in situ* hybridization, to pinpoint their exact sites of action during drought stress.

### 4.4 Implications for drought tolerance breeding and ecological application

The identification of nine *BcSnRK2* genes, particularly the strong and sustained drought inducible characteristics of *BcSnRK2.9* and *BcSnRK2.7* in upper tissues and *BcSnRK2.7* and *BcSnRK2.3* in lower tissues, highlights their promising potential as target genes for genetic improvement of drought tolerance. This potential extends not only to *Bombax ceiba* itself but also to related woody crops. For instance, overexpression of *BcSnRK2.9* or *BcSnRK2.7* in poplar (*Populus* spp.) or eucalyptus (*Eucalyptus* spp.) could potentially enhance water use efficiency and improve shoot level stress tolerance, two key traits associated with drought adaptation in forest trees. On the other hand, natural sequence variations in the promoter regions of these *BcSnRK2* genes across different *Bombax ceiba* ecotypes could be exploited for marker assisted selection (MAS). This approach would facilitate the identification of drought resilient planting materials, which are well suited for reforestation programs in arid and semi-arid regions (Luo et al., 2021; Yasin et al., 2026).

From an ecological perspective, the retention of nine SnRK2 members in *Bombax ceiba*, a typical drought tolerant tropical pioneer tree species, suggests that this gene family size represents an evolutionarily stable configuration that balances functional diversity with regulatory efficiency for drought adaptation in seasonally dry tropical environments. The clear tissue specific expression patterns and distinct temporal drought response profiles among *BcSnRK2* paralogs indicate that functional diversification at the transcriptional level has been a key mechanism enabling this species to cope with water deficit across different organs and stress durations.

Several limitations of this study should be noted. Kinase activity was not measured, as post translational regulation often decouples transcript levels from enzyme function (Lin et al., 2021; Maszkowska et al., 2021). PPI predictions based on *Arabidopsis* homologs require *in vivo* validation in *Bombax ceiba*, and the precise roles of candidate genes such as BcSnRK2.9, BcSnRK2.7, and BcSnRK2.3 need functional testing via overexpression or CRISPR mediated knockout in homologous or heterologous systems. Whether BcSnRK2s participate in ABA independent signaling pathways remains to be fully elucidated (Soma et al., 2020), and the predicted SLAC1 interaction awaits electrophysiological confirmation. Despite these limitations, this study provides fundamental genetic resources and reference data for exploring drought adaptation mechanisms and genetic improvement of woody plants.

## 5 Conclusions

In summary, this study presents the first genome-wide identification and characterization of the *SnRK2* gene family in *Bombax ceiba*, revealing nine members that fall into three conserved phylogenetic clades. These members share conserved kinase domains and exon–intron structures, yet exhibit clear functional divergence, as reflected in their distinct tissue-specific expression profiles and promoter cis-element compositions. Under PEG-simulated drought stress, all nine *BcSnRK2* genes showed tissue- and time-dependent transcriptional responses. Notably, *BcSnRK2.9* and *BcSnRK2.7* displayed strong and sustained activation in shoots, whereas *BcSnRK2.7* and *BcSnRK2.3* responded prominently in roots. Subcellular localization confirmed their distribution in the cytoplasm, plasma membrane, and nucleus, and protein interaction predictions placed them within conserved ABA signaling modules. These findings advance the molecular understanding of drought tolerance in tropical woody plants and provide valuable gene resources for future genetic improvement of stress resistance in trees.

## Data Availability

The original contributions presented in the study are included in the article/**Supplementary Material**. Further inquiries can be directed to the corresponding author.
