# Supplementary material for: Genome-wide identification of the SnRK2 gene family and its response to drought stress in Bombax ceiba
Source: Front Plant Sci. 2026 Jul 7;17:1873223. doi: 10.3389/fpls.2026.1873223 (PMC13385669; doi:10.3389/fpls.2026.1873223)

**
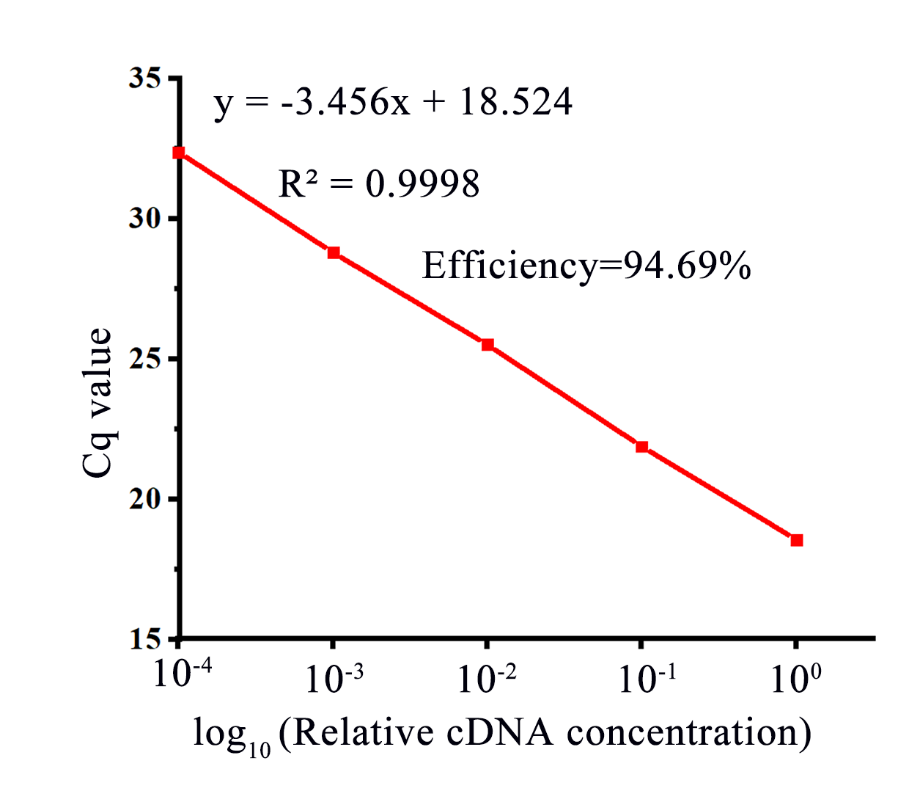
**

**Figure S1. Standard curve of *BcUBQ5* gene by qRT-PCR.**

Ten-fold serial dilutions of cDNA template were amplified using *BcUBQ5*-specific primers. The Cq values were plotted against the log₁₀ of template dilution fold. The linear regression equation and correlation coefficient (R²) are shown on the graph. The amplification efficiency was calculated as [10^-(-1/slope)^ – 1] × 100%.

**
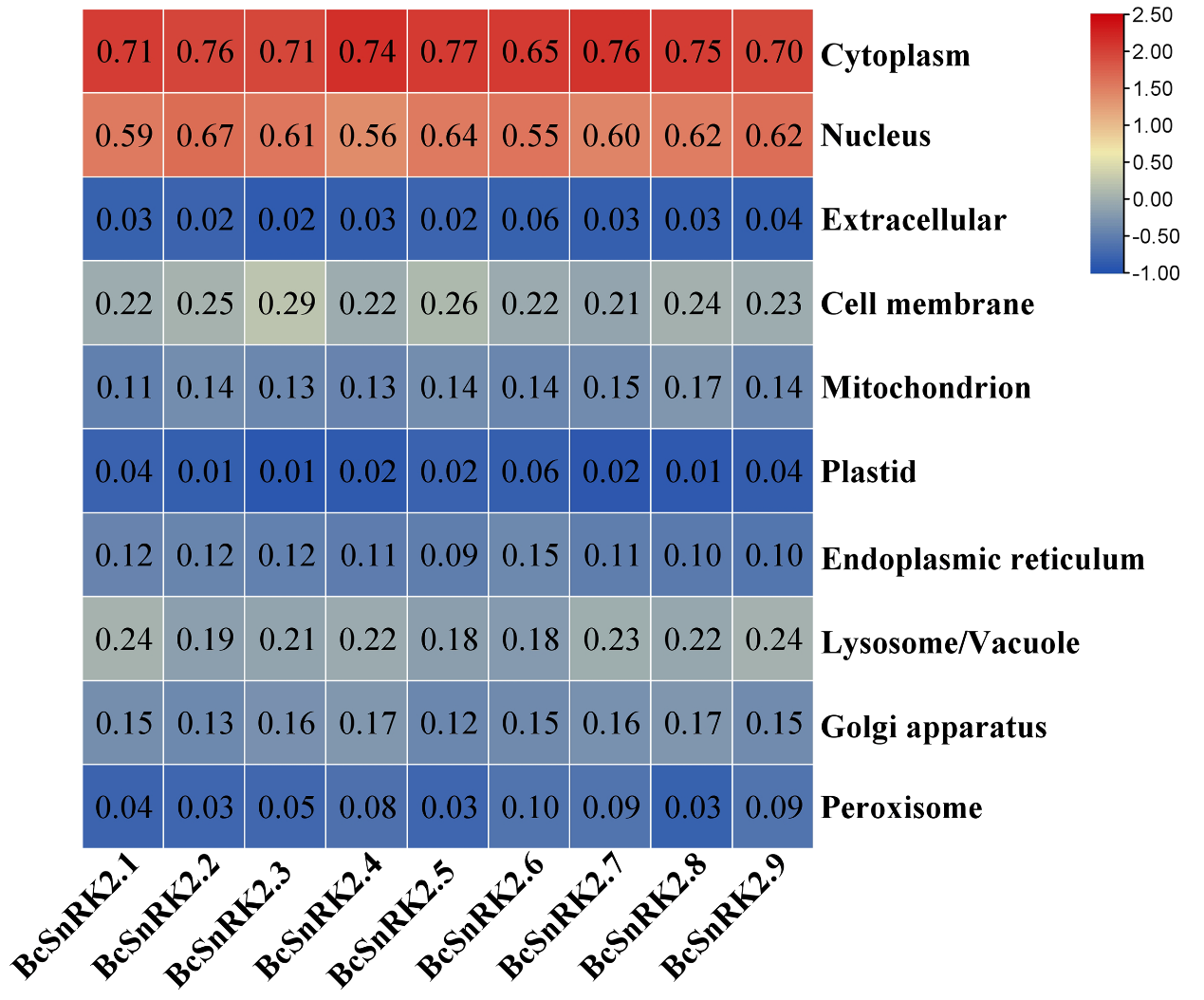
**

**Figure S2.** **Subcellular localization prediction of BcSnRK2 proteins.**

The heatmap illustrates the predicted subcellular localization probabilities of the five BcSnRK2 proteins across different cellular compartments. Each row represents a specific compartment, and each column corresponds to one BcSnRK2 member (BcSnRK2.1 to BcSnRK2.9). The color scale (0.00–0.80) indicates the probability of localization, with red denoting high probability and blue denoting low probability. All BcSnRK2 proteins are predicted to localize primarily to the cytoplasm and nucleus.

**Table S1. All primers used in this study.**

| **Primer name** | **Primer sequence** |
| --- | --- |
| BcSnRK2.1-CDS-GWR-F | GGGGACAAGTTTGTACAAAAAAGCAGGCTTCATGGATCGATCAGCAAAAAC |
| BcSnRK2.1-CDS-GWR-R | GGGGACCACTTTGTACAAGAAAGCTGGGTCTCACATTGCATATATTATCT |
| BcSnRK2.2-CDS-GWR-F | GGGGACAAGTTTGTACAAAAAAGCAGGCTTCATGGAGAAGTACGAGCTGGT |
| BcSnRK2.2-CDS-GWR-R | GGGGACCACTTTGTACAAGAAAGCTGGGTCTTAAGAGATGTGAATTTCTC |
| BcSnRK2.3-CDS-GWR-F | GGGGACAAGTTTGTACAAAAAAGCAGGCTTCATGGAGCGTTATGAGATACT |
| BcSnRK2.3-CDS-GWR-R | GGGGACCACTTTGTACAAGAAAGCTGGGTCTCACACTTGCAATGCACAGA |
| BcSnRK2.4-CDS-GWR-F | GGGGACAAGTTTGTACAAAAAAGCAGGCTTCATGGAGAAGTATGAGGTGGT |
| BcSnRK2.4-CDS-GWR-R | GGGGACCACTTTGTACAAGAAAGCTGGGTCTTACGGCATAGATGCTGGAG |
| BcSnRK2.5-CDS-GWR-F | GGGGACAAGTTTGTACAAAAAAGCAGGCTTCATGGATCGAGCAGACTTGAC |
| BcSnRK2.5-CDS-GWR-R | GGGGACCACTTTGTACAAGAAAGCTGGGTCTTACATTGCGTAGACTATCTC |
| BcSnRK2.6-CDS-GWR-F | GGGGACAAGTTTGTACAAAAAAGCAGGCTTCATGGAGAAGTACGAGCTGGT |
| BcSnRK2.6-CDS-GWR-R | GGGGACCACTTTGTACAAGAAAGCTGGGTCTCAAGCTCTACCCAAGATCAG |
| BcSnRK2.7-CDS-GWR-F | GGGGACAAGTTTGTACAAAAAAGCAGGCTTCATGGATCGTTATGAGATACT |
| BcSnRK2.7-CDS-GWR-R | GGGGACCACTTTGTACAAGAAAGCTGGGTCTCAAACTTGTAATGCACAGAC |
| BcSnRK2.8-CDS-GWR-F | GGGGACAAGTTTGTACAAAAAAGCAGGCTTCATGGACCGTTATGAGATTTT |
| BcSnRK2.8-CDS-GWR-R | GGGGACCACTTTGTACAAGAAAGCTGGGTCTCACAATGGGCACACAAAGT |
| BcSnRK2.9-CDS-GWR-F | GGGGACAAGTTTGTACAAAAAAGCAGGCTTCATGGAGGAGAGGTATGAGCC |
| BcSnRK2.9-CDS-GWR-R | GGGGACCACTTTGTACAAGAAAGCTGGGTCTCACACATAGGCATCATAGTC |
| BcSnRK2.1-qPCR-F | GGTTGATGAGGGACAAGCAGACTG |
| BcSnRK2.1-qPCR-R | GGATGCCTTAGTGACCTGTGGTTG |
| BcSnRK2.2-qPCR-F | CAAGTCAACGGTCGGAACTCCAG |
| BcSnRK2.2-qPCR-R | CCACACGTCGGCCATCTTGC |
| BcSnRK2.3-qPCR-F | CCAGCCTATATTGCGCCTGA |
| BcSnRK2.3-qPCR-R | AAGGATAAGCCCCAACCAGC |
| BcSnRK2.4-qPCR-F | CCGCTTCAAGGAGGTAGTTCTGAC |
| BcSnRK2.4-qPCR-R | GGCACTGCAAATCCGTTCAAAGAG |
| BcSnRK2.5-qPCR-F | TTCGAGCGAATTTGTGCTGC |
| BcSnRK2.5-qPCR-R | TTGCATCGCATGGCAATAGC |
| BcSnRK2.6-qPCR-F | GTTGGTGCCTACCCATTCGA |
| BcSnRK2.6-qPCR-R | TATGCGAGAAAGGAGGTGCC |
| BcSnRK2.7-qPCR-F | GAGGATGGCGACCTGGAAAA |
| BcSnRK2.7-qPCR-R | TCATCGAGATCCATGCTGCC |
| BcSnRK2.8-qPCR-F | CGAACTGACGGAAGGTGGAA |
| BcSnRK2.8-qPCR-R | ATCCCATGGAGAGTTCCCCA |
| BcSnRK2.9-qPCR-F | TCCGACACCGCGTCTTAAAA |
| BcSnRK2.9-qPCR-R | CTTCGGGGGCAATATACGCT |
| BcUBQ5-qPCR-F | CGCACCTTAGCCGACTACAA |
| BcUBQ5-qPCR-R | GGCGAGCTTGACCTTCTTCT |

**Table S2. Full-length protein sequence of BcSnRK2**

| >BceiG003955 BcSnRK2.1 | MDRSAKTVGPGMDMPIMHDSDRYELVRDIGSGNFGVARLMRDKQTDELVAVKYIERGEKIDENVQREIINHRSLRHPNIVRFKEVILTPTHLAIVMEYASGGELFERICNAGRFSEDEARFFFQQLISGVSYCHAMQVCHRDLKLENTLLDGSPAPRLKICDFGYSKSSVLHSQPKSTVGTPAYIAPEVLLKKEYDGKIADVWSCGVTLYVMLVGAYPFEDPEDPKNFHKTIHRILSVQYSIPDYVHISPECRHLISRIFVADPAKRISIPEIRNHQWFLKNLPADLMDENTMNNQFEEPDQPTQSVDEIMQIISEATIPAANTNCLNQYLTGSLDIDDDMEEDLDSDPELDLDSSGEIIYAM* |
| --- | --- |
| >BceiG006852 BcSnRK2.2 | MEKYELVKDIGSGNFGVARLMRNKETKELVAMKYIDRGDKIDENVAREIINHRSLRHPNIIRFKEVVLTPTHLAIVMEYAAGGELFERICNAGRFSEDEARYFFQQLISGVSYCHSMQICHRDLKLENTLLDGSPAPRLKICDFGYSKSSLLHSRPKSTVGTPAYIAPEVFSRREYDGKMADVWSCGVTLYVMLVGAYPFEDQEDPKNFRKTISRIMSVQYKIPDYVHISQDCRHLLSRIFVASPSRRIIIKDIKSHPWFLKNLPRELTEAAQAAYYKKENPTFSLQTVEEIMKIVEEAKVAPPVFPSIGFFGWGGEEGWDTREGDTEQEEDEDEYEKTVKEAHASGEIHIS* |
| >BceiG007606 BcSnRK2.3 | MERYEILKDIGSGNFGVAKLARDKWTGELYAVKYIERGPKIDEHVQREIMNHRSLKHPNIIRFKEVLLTPTHLAIVMEYAAGGELFERICNAGRFSEDEARFFFQQLISGVSYCHAMQICHRDLKLENTLLDGSTAPRLKICDFGYSKSSVLHSQPKSTVGTPAYIAPEVLSRKEYDGKIADVWSCGVTLYVMLVGAYPFEDPEDPRNFRKTIQRILSVHYSIPDYVRLSKDCKHLLSRIFVADPEKRITIPEIKQHPWFLKNLPMEFMEGADGNLGNEKENDQYSQSIEQVLSIIDEARKAGEGAKVGSQLLGGIMDLDDIDADADIDDDIETSGDFVCALQV* |
| >BceiG007389 BcSnRK2.4 | MEKYEVVKDLGAGNFGVARLLRHKDTKELVAMKYIERGHKIDENVAREIINHRSLRHPNIIRFKEVVLTPTHLAIVMEYAAGGELFERICSAGRFSEDEARYFFQQLISGVNYCHFMQICHRDLKLENTLLDGSPAPRLKICDFGYSKSSLLHSRPKSTVGTPAYIAPEVLSRREYDGKLADVWSCGVTLYVMLVGAYPFEDQEDPRNFRKTIQRIMAVHYKIPDYVHVSQDCRNLLSLIFVANPSRRITLKEIKNHPWFLKNLPRELTDTEQASYYQRDNPTFSLQSVDEIMKIVEEARSPPPASMP* |
| >BceiG016110 BcSnRK2.5 | MDRADLTVGPAMDMPIMHDSDRYDFVKDIGSGNFGVARLMRDKVTKELVAVKYIERGDKIDENVQREIINHRSLRHPNIVRFKEVILTPTHLAIVMEYASGGELFERICAAGRFNEDEARFFFQQLISGVSYCHAMQVCHRDLKLENTLLDGSPAPRLKICDFGYSKSSVLHSQPKSTVGTPAYIAPEVLLRKEYDGKIADVWSCGVTLYVMLVGAYPFEDPDEPKDFRKTIQRILSVQYAIPDFVQISPECQHLISRIFVADPTARITIPEIRNHQWFLKNLPADLMDENTMGNHFEEPDQPMQSTDTIMQIIAEATIPAAGLNHYMVENPEDEDMDDLDSESELDVDSSGEIVYAM* |
| >BceiG014682 BcSnRK2.6 | MEKYELVKDIGSGNFGVARLMRNKETKELVAMKYIERGSKIDENVAREIINHRSLRHPNIIRFKEVVLTPTHLAIVMEYAAGGELFERICSAGRFSEDEARYFFQQLISGVSYCHSLQICHRDLKLENTLLDGSPAPRLKICDFGYSKSSLLHSRPKSTVGTPAYIAPEVLSRREYDGKMADVWSCGVTLYVMLVGAYPFEDQEDPRNFRKTISRIMSVQYKIPDYVHISQDCRHLLSRIFVATPSRRITIKEIKSHPWFLKNLPRELTEAAQAAYYRKENPTFSLQSVEEIMKIVEEAKVAPPVSRSIGGFGWGGEEDGDAREEDAEEEEVEEEDEYDKTVKEGVLLLILGRA* |
| >BceiT027085 BcSnRK2.7 | MDRYEILKDIGSGNFGVAKLVRDKWCGELYAVKYIERGPKIDEHVQREIMNHRSLKHPNIIRFKEVLLTPTHLAIVMEYAAGGELFERICNAGRFSEDEARFFFQQLISGVRYCHAMQICHRDLKLENTLLDGSTAPRLKICDFGYSKSSLFHSQPKSTVGTPAYIAPEVLSRKEYDGKIADVWSSGVTLYVMLVGAYPFEDPEDPRDFRKTIQRILSVHYTIPDYVRVSKECRHLLSRIFVANPEMRITISEIKQHAWFLKNLPMEFMEGEEDGDLENEEENCQSQSIEEILSIIDEARKPGEGLKVGSQLLGGGSMDLDDDIDADIDDIETSGDFVCALQV* |
| >BceiG014219 BcSnRK2.8 | MDRYEILKDIGSGNFGVAKLVRDKWTKELFAVKLIERGKKIDEHVQREIMNHRSLKHPNIVRFKEVLLTPTHLAIVMEYAAGGELFERICNAGRFSEDEARFFFQQLISGVSYCHSMQICHRDLKLENTLLDGSTAPRVKICDFGYSKSSVLHSQPKSTVGTPAYIAPEVLSKKEYDGKIADVWSCGVTLYVMLVGAYPFEDPDDPKNFRKTIGRILSVHYSIPDYVRVSVECKHLLSRIFVANPEKRITIPEIRSHPWFLKNLPIELTEGGSWQSHDVNNPSQTMEEVQCIIQEAMKTTEVPKAGELSMGLGSMDLDDLDADADLEDVETSGDFVCPL* |
| >BceiG012468 BcSnRK2.9 | MEERYEPLKELGSGNFGVARLVKDKKTKELVAVKYIERGKKIDENVQREIINHRSLRHPNIIRFKEVLLTPTHLAIVMEYAAGGELFERICSAGRFSEDEARFFFQQLISGVSYCHSMQICHRDLKLENTLLDGSPTPRLKICDFGYSKSAVLHSQPKSTVGTPAYIAPEVLSRKEYDGKIADVWSCGVTLYVMLVGAYPFEDPDDPRNFRKTIGRIMSVQYSIPDYVRVSADCRQLLSRIFVANPAKRISIPEIKQHPWFLKNLPKELVEIEKTNYAESARDQPSQSVEEIMRIIQEAKTPGEGAKVGEQAVAGSSDPNDTEADLESEIDVSGDYDAYV* |

**Table S3.** **Basic information of the *SnRK2* gene family in *Bombax ceiba.***


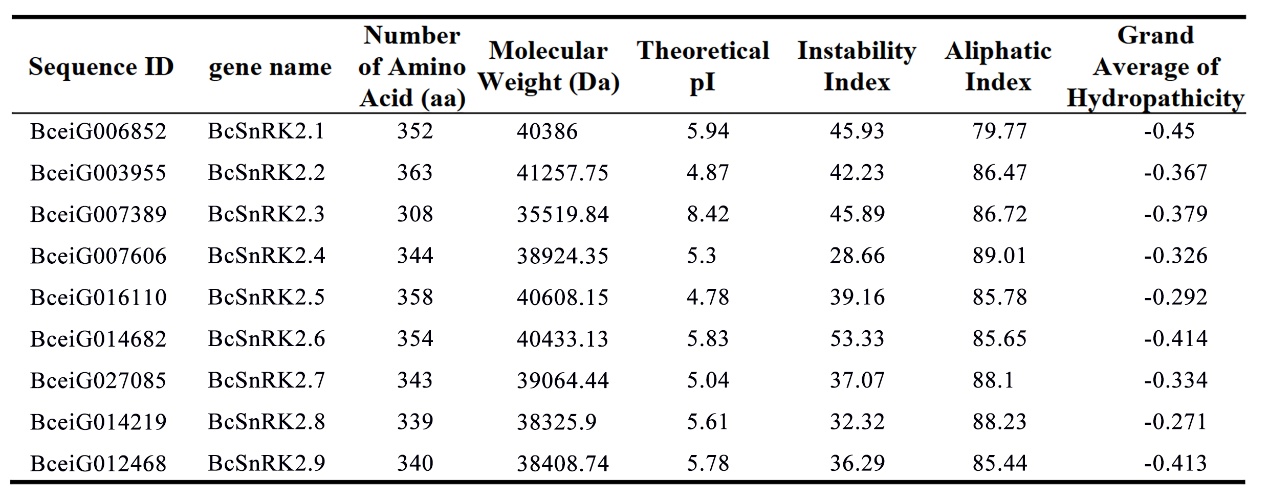

Supplement: Supplementary file 1 [file DataSheet1.docx]
